# Chromosome-scale assembly of yeast genomes using enzyme-free, dense optical genome mapping

**DOI:** 10.1038/s41467-026-78211-7

**Published:** 2026-10-01

**Authors:** Luis Mario Leal-Garza, Hanna Zachrisson, Albertas Dvirnas, Gaurav Goyal, Ikenna Obi, Sriram KK, Nasim Sabouri, Tobias Ambjörnsson, Fredrik Westerlund

**Affiliations:** 1 Department of Life Sciences, Chalmers University of Technology, Gothenburg, Sweden; 2 Department of Mathematics and Mathematical Statistics, Umeå University, Umeå, Sweden; 3 Department of Medical Biochemistry and Biophysics, Umeå University, Umeå, Sweden; 4 Science for Life Laboratory, Umeå University, Umeå, Sweden; 5 Department of Earth and Environmental Sciences, Lund University, Lund, Sweden

**Keywords:** Genomics, Nanobiotechnology, Genetic techniques

## Abstract

Large repetitive elements and complex chromosomal organization continue to challenge accurate assembly of eukaryotic genomes. Optical genome mapping (OGM) provides long-range genomic information by imaging individual DNA molecules. While most OGM protocols rely on sparse enzymatic labeling that restricts resolution in poorly labeled or structurally complex regions, dense labeling strategies generate continuous fluorescence intensity profiles that offer a complementary representation of genome structure. Here we extend our Dense Optical Genome Mapping Assembly (DOGMA) pipeline, previously used for bacterial genomes, to eukaryotic genomes, exemplified by the yeasts *Saccharomyces cerevisiae* and *Schizosaccharomyces pombe*, with compact, yet structurally rich genomes. We use a competitive binding (CB)-based dense OGM protocol that produces continuous intensity profiles, reflecting local AT/GC-content, along individual DNA molecules. By explicitly accounting for structural features inherent to eukaryotic genomes that complicate assembly, DOGMA reconstructs genome-wide optical maps while avoiding collapse of non-adjacent repetitive loci. The resulting chromosome-scale assemblies span the genomes of both yeasts and show local differences relative to the corresponding reference genomes, including expanded repetitive arrays ( > 500 kbp). These results establish a comprehensive framework for scalable dense OGM to increasingly complex genomes, including the human genome, where we foresee that it can be efficiently used for disease diagnosis.

## Abstract (web summary)

Optical genome mapping (OGM) provides long-range genomic information but relies on sparse enzymatic labeling that restricts resolution in poorly labeled or structurally complex regions. Here the authors assemble yeast genomes at chromosome scale using dense OGM, resolving large repetitive regions.

## Introduction

Accurate and complete assembly of eukaryotic genomes remains a fundamental challenge in modern genomics. Despite substantial advances in sequencing technologies, many assemblies continue to suffer from unresolved gaps, collapsed repeats, and structural ambiguities. These limitations are particularly pronounced in regions characterized by long repetitive elements, segmental duplications, and complex chromosomal architectures^1^. While short-read sequencing platforms provide base-level accuracy, they inherently lack long-range information. Third generation long-read technologies extend read lengths into tens of kilobases, but remain susceptible to coverage biases, elevated error rates, and difficulties in resolving highly repetitive or low-complexity regions^2^. As a result, even well-studied eukaryotic genomes often contain poorly resolved regions such as centromeres, telomeres, ribosomal DNA (rDNA) arrays, and subtelomeric regions.

Optical Genome Mapping (OGM) has emerged as a powerful complementary approach for capturing long-range genomic structure. By imaging individual DNA molecules, hundreds of kilobases to megabases in length, OGM provides direct access to genome-wide structural information that is difficult to obtain through sequencing alone. OGM has proven particularly valuable for characterizing large structural variants, copy number changes, and repeat expansions, and has been successfully applied in both research and clinical settings^3–5^. Commercial implementations, such as those by Bionano Genomics, rely on enzymatic labeling of specific sequence motifs, producing sparse fluorescent tag patterns (on average 9–15 per 100 kbp) along DNA molecules^6^. While this approach has demonstrated clinical utility, the relatively low label density limits its ability to resolve certain genomic regions, especially those with low motif frequency or structural complexity, including centromeres and large repeats.

As an alternative to sparse labeling, dense labeling strategies that create continuous intensity profiles have emerged. Throughout this work, we refer to these one-dimensional intensity profiles as barcodes, following terminology established in the OGM community. Historically, the term originated from sparse-label OGM, where fluorescent labels form barcode-like patterns along DNA molecules, and has since been adopted for dense labeling approaches despite the continuous nature of the signal^7^. Some approaches employ DNA methyltransferases with short (4-nt) recognition motifs, resulting in closely spaced labels whose fluorescence signals overlap due to the diffraction limit^8^. Other dense OGM strategies avoid enzymatic labeling altogether, instead using AT/GC-specific non-covalent binding proteins or synthetic dyes, such as the AT-specific TAMRA polypyrrole^9,10^.

A distinct enzyme-free dense labeling strategy developed in our laboratory is based on competitive binding (CB)^11^. CB-based OGM exploits the competitive binding of YOYO-1, a non-specific fluorescent DNA bis-intercalator, and netropsin, a non-fluorescent molecule with binding specificity for AT-rich regions, to DNA^11^. This interaction produces a continuous fluorescence intensity profile along each DNA molecule, where the signal strength reflects the underlying GC content. While this method cannot resolve individual fluorophores, it generates information-dense, sequence-dependent intensity profiles (barcodes) that provide a continuous representation of the genome compared to motif-based OGM approaches. These barcodes can then be aligned to reference genomes or used for de novo genome assembly^12–14^. Since the fluorescence signal in CB-based OGM arises from YOYO-1 labeling of the DNA backbone, a dye already widely used in OGM and single-molecule assays^15^, CB can be implemented without major modifications to existing instrumentation. This compatibility creates opportunities to integrate CB-based OGM with complementary labeling strategies, enabling multimodal analysis of genomic features along individual DNA molecules.

To exploit the full potential of CB-based OGM, we have recently developed DOGMA (Dense Optical Genome Mapping Assembly), a computational pipeline designed for de novo assembly of densely-labeled genomes, with CB-derived barcodes as application^14^. DOGMA was initially validated on *Escherichia coli*, a bacterial genome characterized by limited redundancy, low large-scale compositional variation, and relatively uniform sequence organization, providing an ideal test for overlap accuracy and assembly contiguity.

Extending DOGMA to eukaryotic genomes introduces a complex set of challenges. In contrast to *E. coli*, even relatively small eukaryotic genomes contain complex features, including extensive repetitive regions and large-scale compositional domains that severely impede both sequence- and OGM-based assembly methods^1,16^. Repetitive elements often exceed sequencing read length and occur at multiple loci, making it difficult to assign reads or barcodes to unique genomic positions. Large-scale compositional domains, such as extended AT-/GC-rich regions or gradual changes in AT/GC content, further reduce barcode uniqueness by producing similar intensity profiles across long genomic regions. In OGM, both types of features can generate similar barcode patterns at distant loci, leading to ambiguous placement during alignment and assembly. Examples of these features include rDNA arrays, centromeres, telomeres, and subtelomeric regions, which are structurally complex, highly repetitive, and often appear at multiple loci in a genome^17–20^. As a result, these regions are systematically underrepresented or misassembled in reference genomes.

In this study, we extend DOGMA to assembly of eukaryotic genomes using *Saccharomyces cerevisiae* and *Schizosaccharomyces pombe* as model systems. These two yeasts provide complementary test cases for CB-based OGM due to their compact, yet structurally rich, genomes and distinct chromosomal architectures. In *S. cerevisiae*, regions such as the rDNA array on chromosome XII are associated with genome stability, cellular aging, and nucleolar organization and are notoriously difficult to resolve^21–23^. Likewise, in *S. pombe*, similar arrays located near both ends of chromosome III pose challenges during DNA replication and remain incompletely resolved by sequencing-based approaches, consistent with their repetitive structure^24,25^. Similar repetitive structures are widespread in larger eukaryotic genomes.

We use DOGMA here to generate de novo OGM assemblies of eukaryotic genomes that capture key genomic features, including telomeric regions and large repetitive arrays, while explicitly examining regions where assembly contiguity breaks down. We subsequently use reference-based analysis for refinement of unresolved joins (genomic regions covered by at least two adjacent contigs), validation and biological interpretation. With these results we demonstrate the feasibility of dense OGM assembly in eukaryotes and the biologically driven challenges that must be addressed when extending dense OGM-based assembly methods beyond bacterial genomes.

## Results

**Fig. 1:**
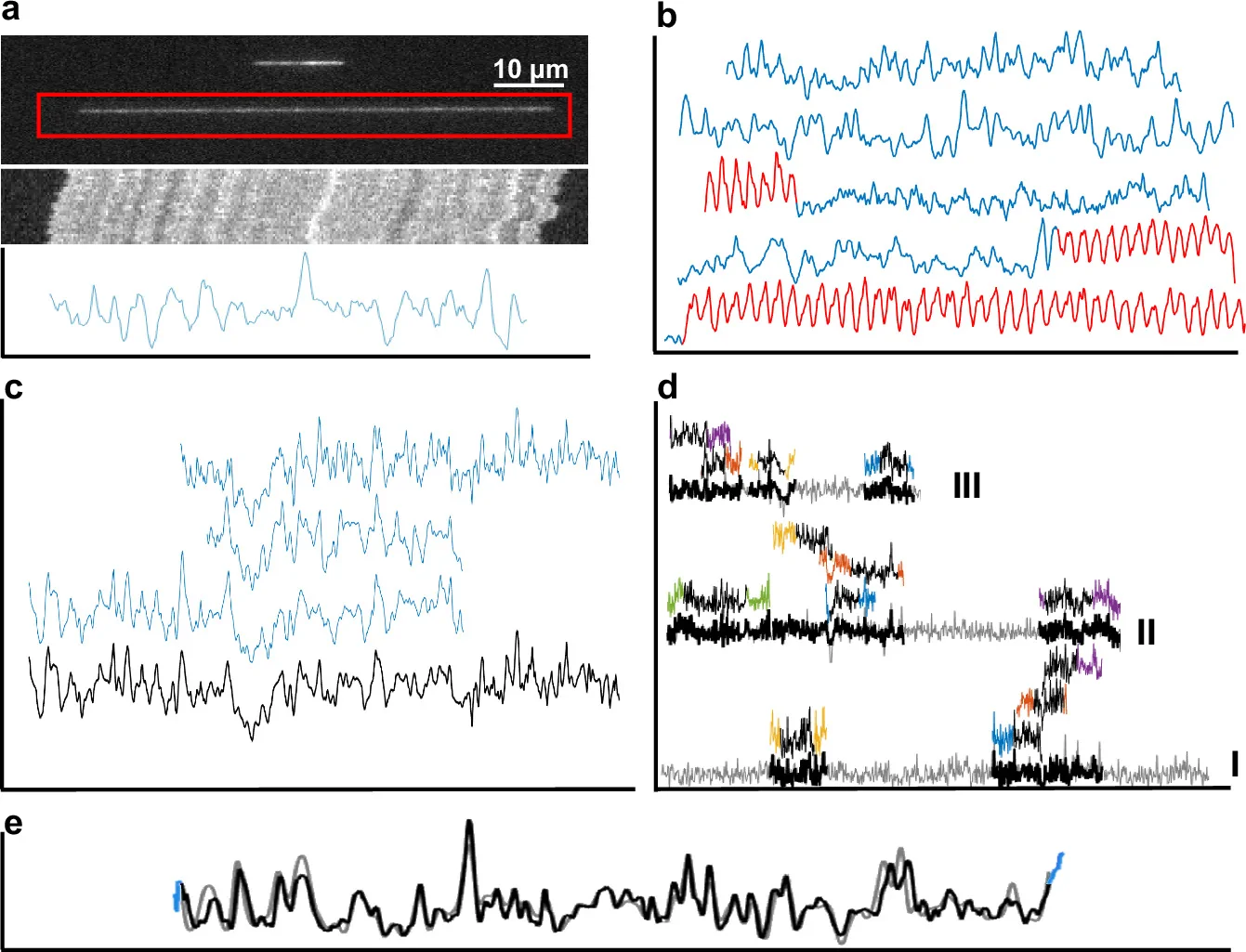
Workflow overview. **a** CB-labeled DNA molecules are stretched in nanochannels, imaged and detected (red box) to generate continuous fluorescence intensity profiles, referred to as barcodes. **b** Extracted barcodes undergo automated quality control and repetitive region (red) detection. **c** Multi-step assembly with the DOGMA pipeline generates barcode contigs (bargroups, black) from individual barcodes (blue). **d** Bargroups (colored) are aligned to reference genomes (gray) for reference-assisted refinement of unresolved joins, validation and structural comparison. Roman numerals indicate chromosome numbers. **e** Chromosome-scale assemblies (black) are generated from consensus bargroups and aligned to each reference chromosome (gray). Segments present in the reference but not in the consensus are highlighted in yellow and segments present in the consensus but not in the reference in blue. Source data are provided as a Source Data file.

To perform OGM, we stained genomic DNA samples and stretched them by confinement in nanofluidic devices. Details of the experimental and computational workflows are described in Methods. In brief, DNA molecules were extracted from yeast cells enclosed in agarose plugs, labeled by competitive binding of YOYO-1 and netropsin, extended in nanochannels, and imaged by fluorescence microscopy, producing continuous intensity profiles along individual DNA molecules. Images were then processed to extract one-dimensional fluorescence intensity profiles, referred to as barcodes (Fig. 1a). The extracted barcodes underwent automated quality control (see Supplementary Methods 1) and repetitive region screening prior to assembly (Fig. 1b). Assembly was carried out using a multi-step implementation of the DOGMA pipeline^14^, grouping similar barcodes into consensus optical mapping contigs, hereafter referred to as *bargroups*, following the terminology introduced in the DOGMA framework (Fig. 1c). The resulting bargroups were then aligned to the corresponding reference genomes and used for reference-assisted refinement of unresolved joins, while also enabling subsequent validation and structural comparison (Fig. 1d). Finally, refined chromosome-scale assemblies were generated and compared to the corresponding reference chromosomes (Fig. 1e).

### Reference-based alignment

**Fig. 2:**
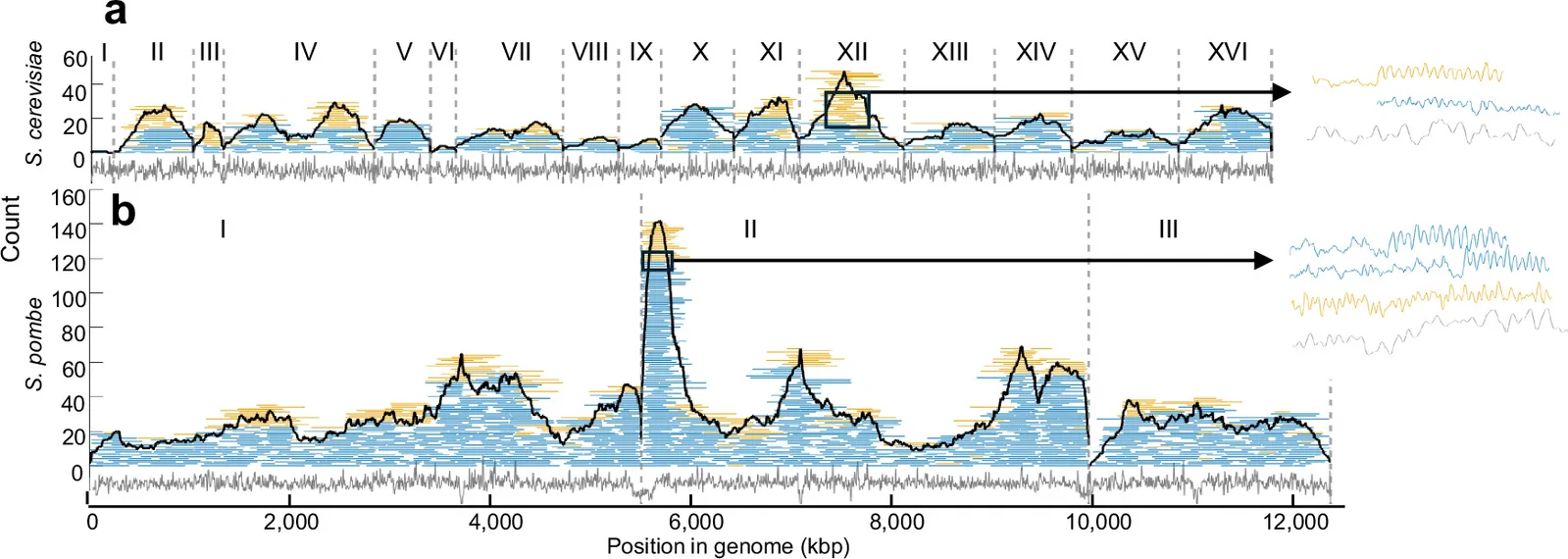
Alignment to reference genomes. Alignment of all barcodes (*N* = 1653) to the reference genomes of **a** *S. cerevisiae BY4742* and **b** *S. pombe 972h-*, with chromosomes indicated as Roman numerals. Matches to the correct species are represented in blue and to the incorrect species in yellow. The theoretical optical maps are shown in gray. Black lines represent coverage depth. Insets highlight the most enriched genomic loci for each organism. Source Data and Source Code underlying the plots in this figure are available on Zenbodo.

A total of 1102 *S. pombe* and 551 *S. cerevisiae* CB-labeled DNA molecules were imaged and converted into barcodes. To evaluate genome-wide coverage prior to the de novo assembly, the barcodes were aligned to theoretical reference barcodes derived from the corresponding reference genomes (see Methods)^26,27^. The alignment was performed using a full-length alignment strategy, in which each query barcode is aligned in its entirety to the reference, with the Pearson correlation coefficient (CC-score) scoring method (Fig. 2, see Methods for details).

Genome-wide alignments were obtained for all three *S. pombe* chromosomes and all sixteen *S. cerevisiae* chromosomes (Fig. 2). Reference-based coverage exceeded 98% for both genomes, with mean coverage depths of 29.5x for *S. pombe* and 16.4x for *S. cerevisiae*, calculated for molecules that passed quality control and were successfully aligned to the respective reference genomes, indicating that the dataset provides near-complete genome representation for downstream assembly.

Despite the general overall uniformity in coverage, several genomic regions, particularly in *S. pombe*, displayed elevated coverage. Given the random sampling of DNA inherent to OGM, pronounced local enrichment is not expected. Elevated coverage is more likely to reflect alignment ambiguity, where barcodes from distinct loci exhibit high full-length similarity and are therefore assigned to alternative genomic positions. Such ambiguity can arise when regions at different loci share similar intensity profiles, or when sequences are incompletely represented in the reference assembly, leading to misplacement.

An additional source of barcode misplacement was observed in specific regions of the *S. cerevisiae* genome, such as chromosome I and VI, where reduced coverage was observed. Inspection of these regions indicated that this localized reduction was associated with a limitation of the full-length alignment strategy, which requires query barcodes to be shorter than the reference. Molecules approaching or exceeding full chromosome length are therefore unable to align optimally, and are instead placed at alternative loci, contributing both to apparent depletion at their true origin and enrichment elsewhere.

As an additional specificity control, barcodes from each species were aligned against the reference genome of the other species (Fig. 2). While a majority of the barcodes (76.4%) aligned preferentially to their correct species, a subset exhibited comparable or higher CC-scores to regions in the reference of the other species. After inspecting cross-species matches (Fig. 2, insets), we noticed that they occurred mainly around specific loci for each genome, creating alignment ambiguity hotspots. We also observed that cross-species matches were enriched for barcodes characterized by either long-range (200–300 kbp) low-frequency intensity trends or extended repetitive regions, both of which reduce barcode uniqueness and increase alignment ambiguity.

Overall, the reference-based analysis indicated that global alignment provides reliable genome-wide coverage estimates, but that it is susceptible to alignment ambiguity under specific structural conditions. Since both reference alignment and DOGMA bargroup formation rely on correlation-based similarity scores (CC-scores), analysis of reference alignment provides an interpretable proxy for understanding sources of ambiguity that may also affect barcode grouping during de novo assembly. To clarify the origin of these effects and assess their impact on assembly, we next examined experimental and theoretical barcode properties influencing misalignment prior to initializing the de novo assembly.

### Genomic features driving alignment ambiguity in eukaryotic OGM

Analysis of the barcode dataset revealed three properties that influence dense OGM analysis: chromosome-spanning molecules, repetitive barcode components, and long-range intensity trends. These properties are examined separately in the following sections. Chromosome-spanning molecules primarily affect reference alignment strategies, whereas repetitive barcode components and long-range intensity trends influence correlation-based similarity scoring and barcode grouping during assembly. We define long-range intensity trends as broad, low-frequency variations in the continuous fluorescence intensity profile extending over hundreds of kilobases, distinct from the local barcode features that provide locus-specific information. Their origin, relationship to genome composition, and impact on similarity scoring are examined in a dedicated section below.

#### Chromosome-spanning molecules are captured by OGM

Using the full-length alignment framework, molecules exceeding the theoretical chromosome length cannot be properly aligned, leading to misplacement or exclusion. This effect was particularly evident for the smaller chromosomes in *S. cerevisiae*, where full-length molecules exceed the corresponding theoretical barcode length.

**Fig. 3:**
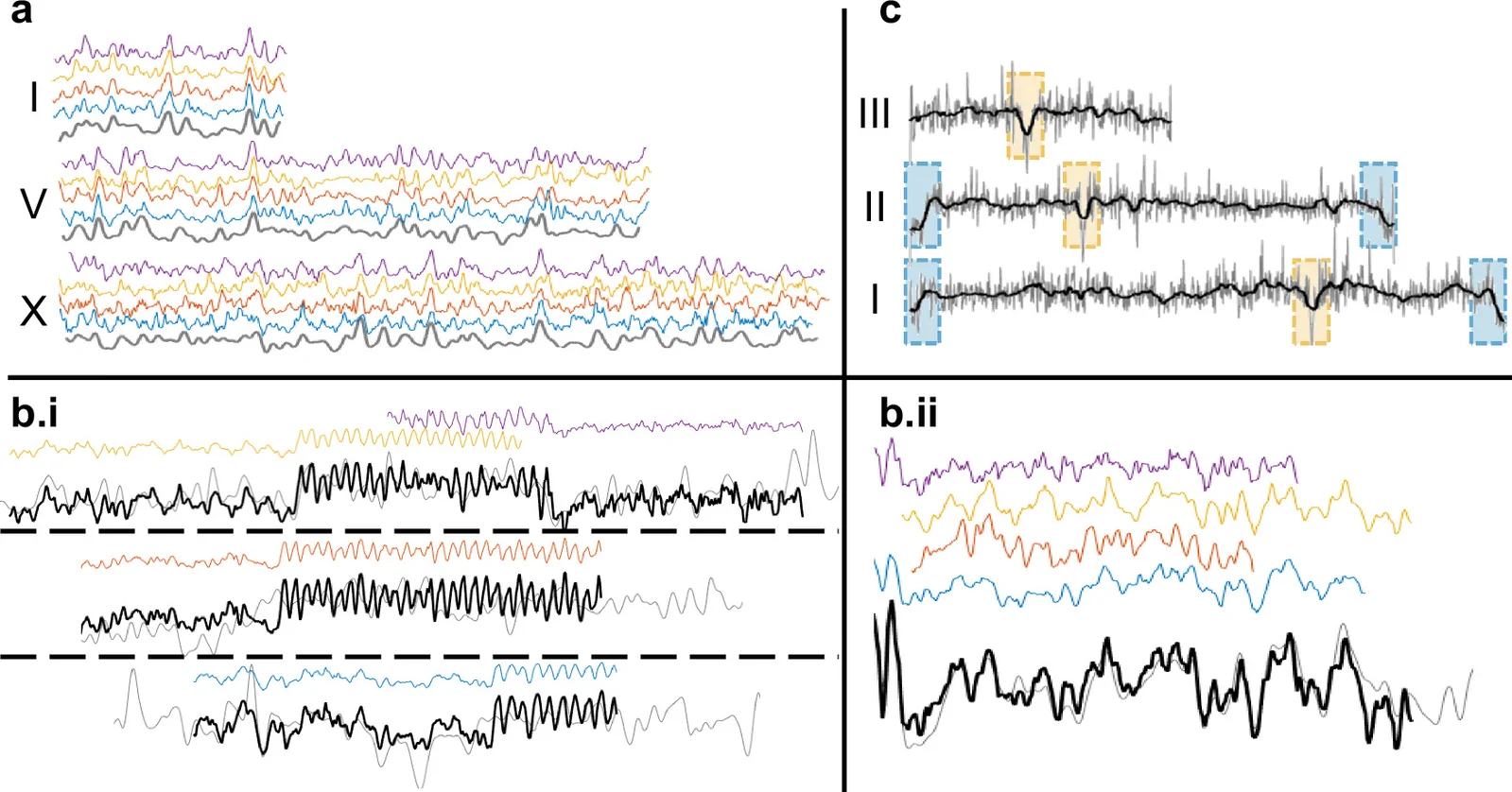
Considerations for dense OGM assembly of eukaryotic genomes. **a** Representative barcodes (colored) spanning entire reference chromosomes (gray) in *S. cerevisiae*. Examples of the same partially repetitive barcodes (colored) aligned to the reference genome (gray) before (**b**.**i**) and after (**b**.**ii**) masking of repetitive regions. Corresponding barcodes have the same color in both panels. The consensus barcode of each alignment is shown in black for comparison. **c** Theoretical barcodes for the three *S. pombe* chromosomes (in gray) and their smoothened trends (black). Orange boxes mark centromeres, showing conserved long-range low-intensity motifs, and blue boxes indicate regions with intensity profile gradients, due to extended GC-content gradients toward chromosome ends. Source data are provided as a Source Data file.

Individual barcodes, up to 800 kbp in size, fully spanned ten of the sixteen *S. cerevisiae* chromosomes (examples in Fig. 3a), demonstrating the presence of chromosome-scale molecules in the dataset. This observation highlights the capacity of CB-based OGM to capture entire eukaryotic chromosomes within single intact DNA molecules. For sufficiently small chromosomes, and given limited DNA fragmentation, assembly complexity may also be reduced since individual molecules can span an entire chromosome. Here, local alignment (see Methods), which identifies the highest-scoring subregions between barcode and reference without requiring full-length matching, is primarily used during reference-based analysis of assembled bargroups, including their placement for subsequent refinement. This strategy is motivated by the observation that experimental molecules can exceed the length of the theoretical reference, making full-length alignment inappropriate for both chromosome-spanning molecules and assembled bargroups.

#### Repetitive barcode components cause alignment ambiguity

A subset of barcodes exhibited broad alignment across multiple genomic loci and were identified as being enriched in repetitive features. The identified repetitive units ranged between 9 and 10.5 kbp in size, consistent with known repetitive regions in *S. cerevisiae* and *S. pombe*, respectively. This repetitive DNA is known to be underrepresented or collapsed to just two copies per locus in current reference assemblies^27,28^.

When repetitive regions are not masked, their repetitive components dominate the alignment, producing visible misalignments to incorrect loci (Fig. 3b.i). To address this issue, and to incorporate repetitive DNA into the alignment and assembly more effectively, we developed a repetitive-element detection algorithm (see Supplementary Methods 2) that identifies and masks repetitive elements. The algorithm also classifies barcodes as fully repetitive, partially repetitive (when a subregion of the barcode has at least five contiguous repetitive units), and non-repetitive.

After masking the repetitive segments, the remaining non-repetitive portion of the barcode can be used to determine the correct genomic locus. As illustrated in Fig. 3b.ii, masking resolves the ambiguity and enables consistent alignment of the same molecules to a single genomic locus. Since reference alignment and DOGMA assembly rely on the same correlation-based scoring framework, the effects of repetitive masking on alignment provide a useful proxy for its expected influence on bargroup formation during assembly.

#### Long-range GC-dependent intensity gradients influence barcode similarity

The reference-based analysis revealed recurrent cases in which barcodes from distinct genomic loci exhibited high CC-scores. Inspection of these barcodes suggested the presence of gradual, long-range intensity variations in addition to the typical CB-based OGM barcode features. To better understand the origin of this behavior, we searched for long-range intensity trends in the theoretical reference barcodes.

Smoothing of theoretical reference barcodes using a moving-average filter revealed pronounced long-range intensity trends spanning hundreds of kilobases in several locations (Fig. 3c), reflecting genome-scale compositional domains that can be detected by dense CB-based labeling. These long-range trends closely follow broad AT/GC compositional sequence variation along the chromosomes, producing smooth intensity fluctuations over hundreds of kilobases. In *S. pombe*, these features were particularly evident, with gradually decreasing GC-content extending ~300 kbp toward chromosome ends and characteristic low-GC motifs spanning ~200 kbp at centromeric regions. While local barcode structure is defined by short-scale intensity variations (5–20 kbp), these long-range trends introduce an underlying “low-frequency” barcode component across much larger genomic distances. To better illustrate this phenomenon, in Supplementary Fig. 4 we show representative barcodes from different loci of the *S. pombe* reference assembly and decompose them into their long-range and local components. There, we demonstrate that long-range intensity trends systematically increase CC-scores between unrelated barcode regions. Consequently, these trends reduce the specificity of similarity-based ranking used during both reference alignment and barcode grouping.

Consistent with this organization, experimental barcodes exhibiting similarly shaped low-frequency fluorescence intensity profiles frequently produced ambiguous alignments, characterized by small differences between the highest- and second highest-scoring matches (low ∆CC-scores). In some cases, comparable scores were also obtained when matching to the reference genome of the other species (Fig. 2).

These results demonstrate that CB-based OGM captures biologically meaningful genome-scale compositional information that is largely inaccessible to sparse labeling approaches that just label specific motifs. While these long-range intensity trends can complicate correlation-based alignment and assembly of dense barcodes, they also capture chromosome-scale compositional sequence variation that is largely inaccessible to sparse labeling approaches, highlighting both a limitation and a unique strength of dense OGM.

To further investigate whether the elevated coverage observed in Fig. 2 reflects intrinsic large-scale compositional features of the genome, rather than coverage limitations, intragenomic self-similarity analysis was performed across the *S. pombe* chromosomes (Supplementary Fig. 5). Chromosomal regions ≥150 kbp exhibiting CC-score ≥0.75 were considered highly similar. This analysis revealed discrete similarity “hotspots” shared between chromosomes. Inspection of these regions showed that many either coincide with, or lie in close proximity to, genomic regions exhibiting pronounced long-range intensity trends (Fig. 3c). In our data, we have observed that these long-range features dominate correlation-based similarity measures despite divergent local structure, providing a mechanistic explanation for the coverage inflation and reduced alignment confidence observed in the reference-based analysis.

Together, these findings indicate that genome-scale sequence features, particularly repetitive regions and long-range compositional sequence variation, rather than insufficient coverage, are the primary source of alignment ambiguity and represent the primary challenges for dense OGM assembly for the yeast genomes analyzed here.

#### Molecule characteristics before and after barcode processing

Mean molecule lengths prior to barcode processing were 337.8 kbp for *S. pombe* and 359.7 kbp for *S. cerevisiae*. Barcode processing included stitching molecules spanning multiple imaging fields, and masking barcode regions dominated by repetitive features (see Methods). Repetitive segments were masked rather than removed, preserving the non-repetitive parts for downstream analysis. Quality-control (QC) filtering additionally removed molecules failing shrink-based criteria (Supplementary Methods). QC parameters were adopted from our previously validated dense OGM pipeline and are described in detail in the Methods and Supplementary Methods. Following preprocessing and QC filtering, the mean molecule lengths remained largely unchanged (337.6 kbp for *S. pombe* and 366.0 kbp for *S. cerevisiae)*, indicating that these steps primarily addressed problematic artifact-prone regions without introducing size-related bias.

To further assess reference-alignment specificity after accounting for chromosome-spanning molecules and repetitive regions, a second reference-based alignment was performed (Supplementary Fig. 3a, b). Local barcode-to-reference matching was used to accommodate chromosome-spanning molecules, while repetitive regions were masked to reduce alignment ambiguity arising from repeated sequences and their underrepresentation in the reference genomes. The resulting alignments were then used to evaluate whether residual alignment ambiguity could contribute to the observed non-uniform coverage. For this purpose, a ∆CC threshold was applied to filter barcodes with ambiguous alignments. ∆CC represents the difference between the highest and second highest CC-scores. Increasing the ∆CC threshold improved organism-specific alignment accuracy while reducing the fraction of retained barcodes, illustrating the trade-off between specificity and molecule retention (Supplementary Fig. 3c). An empirically selected ∆CC threshold of ∆CC = 0.03 was chosen as a compromise between specificity and barcode retention, ensuring high alignment accuracy while maintaining a near-complete estimated coverage of both genomes. This ∆CC threshold was used only for the reference-based alignment analysis and was not applied during the de novo bargroup formation.

### Bargroup formation and consensus barcode construction

**Fig. 4:**
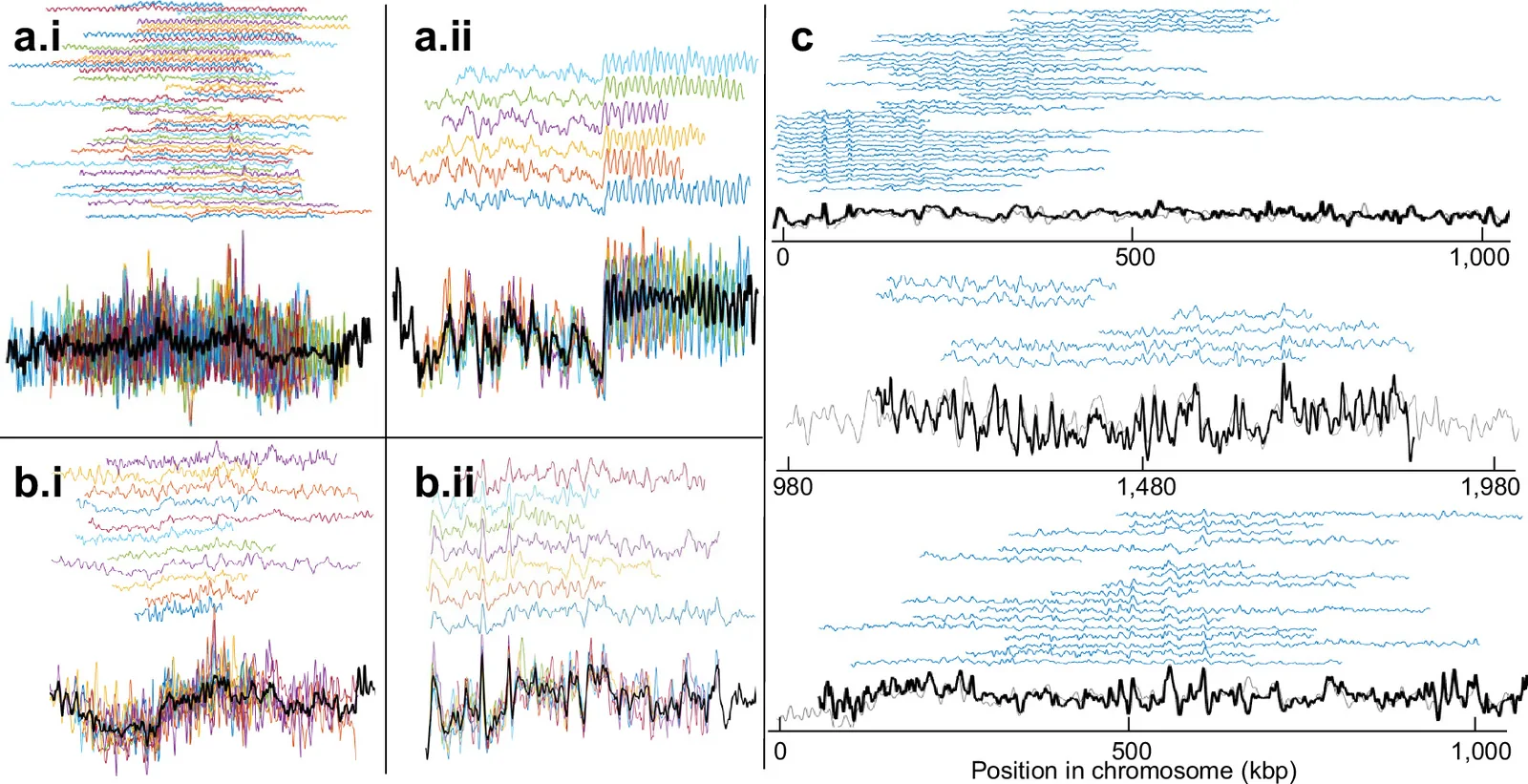
Assembly of bargroups. Example bargroup assembled **a**.**i** without masking and **a**.**ii** after masking of repetitive regions. Constituent barcodes are shown in color and the resulting bargroup consensus in black. Note that the repeat-masking procedure makes it so that the bargroups shown in these examples are assembled from different initial conditions and therefore do not contain the same constituent barcodes. **b** Examples of bargroups illustrating different signal-to-noise (SNR) characteristics. **b**.**i** A bargroup that fails (SNR = 0.9) the SNR criterion and **b**.**ii** a bargroup that passes (SNR = 1.4) the SNR criterion. **c** Example of assembled bargroups aligned to the reference genome. Consensus bargroups (black) constructed from multiple barcode members (blue) are aligned post-assembly to their best matching regions along the theoretical reference barcodes (gray) in chromosomes I, I and II (from top to bottom respectively of *S. pombe*). Source data are provided as a Source Data file.

Having characterized the genomic features influencing similarity metrics and assessed the contribution of alignment ambiguity to the reference-based coverage, we next assembled QC-filtered and repeat-masked barcodes into bargroups using an updated DOGMA pipeline. In our updated pipeline, repetitive elements were identified and masked from barcodes prior to assembly. Assembly without masking produced bargroups containing misaligned repetitive and non-repetitive segments (Fig. 4a.i). Masking allowed the non-repetitive parts of barcodes to drive the assembly, while still maintaining the repetitive content for post-assembly analysis. This strategy enabled correct assembly of molecules that would otherwise be mis-grouped or left ungrouped, while also resolving incorrect groupings driven by repetitive segments (Fig. 4a.ii).

To mitigate grouping driven primarily by shared long-range intensity trends rather than local structural similarity, we introduced a bargroup scoring metric based on signal-to-noise (SNR) characteristics (see Methods). This metric penalizes bargroups dominated by smooth, low-frequency signal components with constituting barcodes that differ in their local features (Fig. 4b.i) and prioritizes bargroups with well-defined local features consistent among their constituting barcodes (Fig. 4b.ii).

Bargroup assembly applied pairwise similarity constraints incorporating limited stretching variation, positional tolerance in pixel coordinates, and statistical thresholds on correlation significance (see Methods). The assembled bargroups were subsequently aligned to the corresponding reference genomes using a local bargroup-to-reference alignment strategy to assess genome-wide coverage and contiguity and to guide subsequent refinement of unresolved joins. Each bargroup consensus was represented as an averaged barcode intensity profile derived from its constituent barcode members (Fig. 4c).

Overall, approximately 20% and 30% of the barcodes used as input for the bargrouping process contributed to bargroup formation from *S. pombe* and *S. cerevisiae*, respectively, while the rest remained ungrouped despite passing QC. Ungrouped molecules were retained for downstream coverage analysis and gap resolution.

### Genome-wide assemblies reveal chromosomal extensions

Genome-wide coverage by assembled bargroups closely mirrored the high reference-alignment coverage observed prior to assembly, indicating that most chromosomal regions were represented within contiguous optical maps. Across multiple chromosomes, bargroups spanned megabase-scale intervals, demonstrating robust large-scale assembly performance.

Following initial assembly, bargroups were aligned to the reference genomes using a local bargroup-to-reference alignment strategy. The resulting reference placements were used to identify unresolved joins between bargroups that could not be resolved during the initial de novo assembly. Adjacent bargroups with consistent placements were subsequently merged based on local bargroup-to-bargroup similarity to generate chromosome-scale assemblies. The resulting assemblies retained only bargroups satisfying the meanCC, stdCC, and SNR quality criteria defined during bargroup formation (Supplementary Table 3). A quantitative comparison of chromosome-level assembly metrics before and after applying these strategies is presented in Supplementary Table 4.

**Fig. 5:**
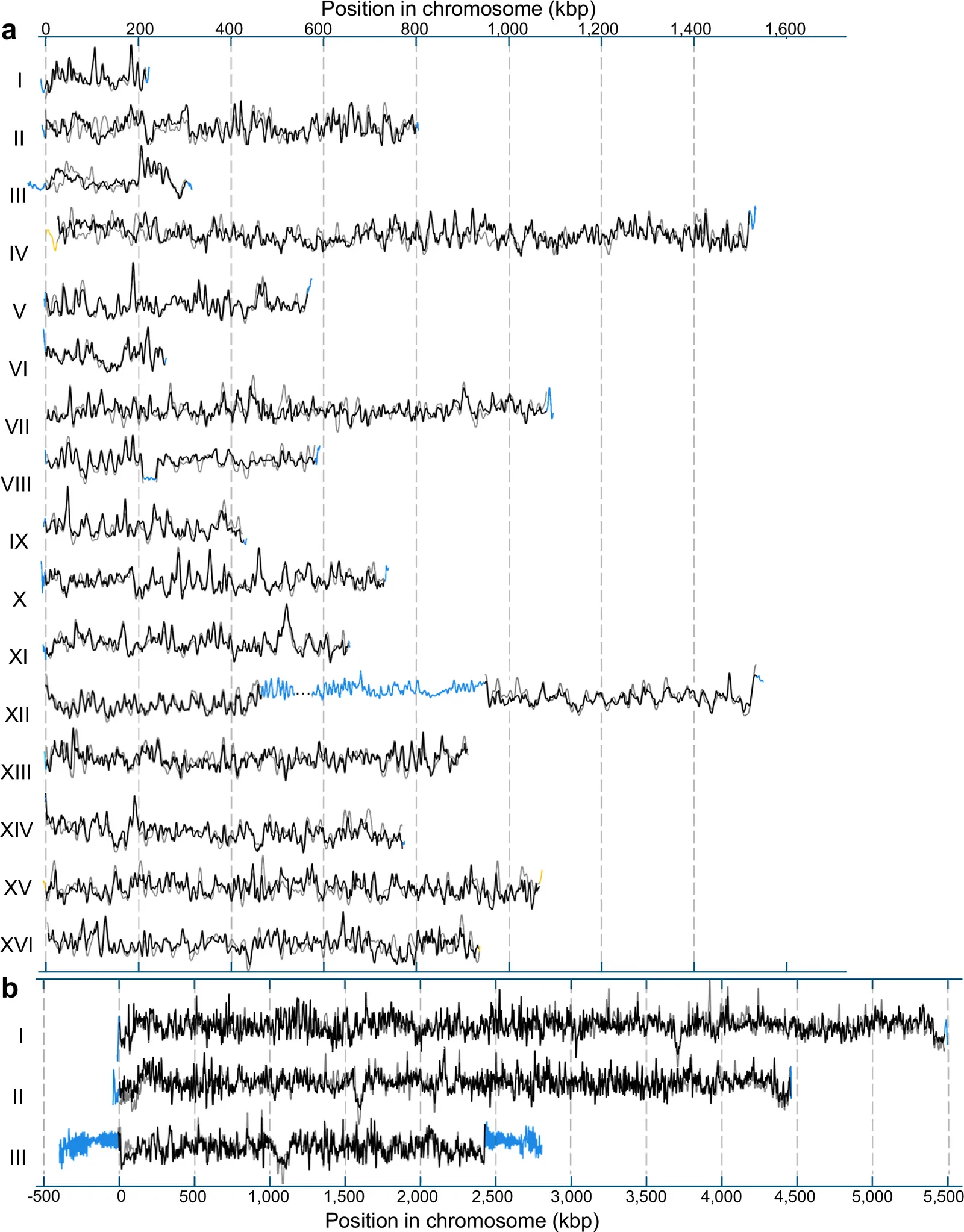
Full genome assemblies. **a** All assembled *S. cerevisiae* chromosomes (black) aligned to the reference (gray). **b** All assembled *S. pombe* chromosomes (black) aligned to the reference (gray). Chromosomal extensions relative to the reference are highlighted in blue and regions not covered by the assembly are highlighted in yellow. **a**, **b** have different size scales for their x-axes. Source data are provided as a Source Data file.

The final assemblies generated for both *S. pombe* and *S. cerevisiae* included highly repetitive regions typically underrepresented in sequence-based assemblies (Fig. 5). Some of the assembled chromosomes show minor extensions (>6 kbp) relative to the reference chromosomes in the end(s) of each chromosome. One potential contributor to these differences is the treatment of barcode boundaries during theoretical barcode generation, where the first and last 6 pixels are excluded because edge intensities are considered less reliable.

Two local breaks in contiguity were observed at *S. pombe* chromosome I from position 4466 kbp to 4787 kbp and chromosome II from position 3208 kbp to 3211 kbp (Supplementary Fig. 6). To distinguish assembly limitations from absence of coverage, we examined high-confidence barcodes that aligned strongly to the reference and that had low alignment ambiguity (∆CC > 0.03), but did not group into bargroups. Inclusion of these molecules revealed that regions lacking assembled contigs were nevertheless supported by individual barcodes, allowing the local contiguity to be restored and resulting in near-complete genome-wide molecular representation.

Large repetitive structures were resolved in both organisms. In *S. cerevisiae*, a large repetitive block interrupted the reference assembly of chromosome XII near 479 kbp. This region extended ≥550 kbp and consisted of ~9 kbp repeat units, corresponding to ≥60 tandem copies. In *S. pombe*, extended repeat arrays were observed at both ends of chromosome III beyond the length represented in the current reference assembly, spanning at least 1 Mb in total (≥500 kbp from each telomeric end). Individual repeat units measured ~10.5 kbp, consistent with rDNA repeats^20,28^ and indicating the presence of ≥50 tandem copies at each end. These observations are consistent with prior PFGE-based studies that have reported substantial variability in rDNA cluster size in both *S. cerevisiae* chromosome XII and *S. pombe* chromosome III, including estimates of >100 rDNA repeat units and chromosome-length variation driven by rDNA copy number changes^29^. This supports the interpretation that these regions correspond to highly expanded rDNA arrays that are incompletely represented in current reference assemblies.

Discrepancies between our assemblies and the *S. cerevisiae* chromosomes VIII and III were also observed. Manual inspection of the alignments between our assemblies and the reference (Fig. 5) suggests an additional ~30 kbp of contiguous sequence in chromosome VIII near position 206 kbp. An inconsistency of similar size was observed on chromosome III between positions 100 kbp and 200 kbp. Since SV detection is not currently integrated into the DOGMA workflow, the structure and breakpoints of these additional regions was not determined directly. Comparison with publicly available long-read assemblies for *S. cerevisiae* S288C^30^ revealed substantially improved agreement with the DOGMA assemblies in both cases (Supplementary Fig. 7), suggesting that these discrepancies may reflect incompletely resolved or collapsed regions in the current reference assembly rather than novel strain-specific variants.

## Discussion

This work demonstrates that dense optical genome mapping (OGM) assembly using the DOGMA pipeline can be extended from bacterial to eukaryotic genomes. In doing so, it reveals constraints and opportunities that are largely invisible in simpler systems, such as bacterial genomes, and require targeted methodological adaptations. Applying DOGMA to *S. cerevisiae* and *S. pombe* demonstrates that OGM-based assembly of eukaryotic genomes is constrained less by genome size and more by repeat composition and long-range genome organization.

For *E. coli*, DOGMA achieved nearly complete genome reconstruction with minimal preprocessing, reflecting the relative simplicity and low redundancy in bacterial genomes^31^. The transition to *S. cerevisiae* and *S. pombe* revealed a fundamentally different assembly landscape. Yeast genomes introduced an increase in barcode ambiguity due to repetitive genomic features such as rDNA arrays and subtelomeric repeats^22,23,26,29^.

By explicitly identifying and isolating repetitive signal components, DOGMA can assemble repeat-rich regions without collapsing non-adjacent loci and subsequently reintegrate them using unique flanking context. Assembly of these repetitive regions with OGM is solely limited to the size of the isolated DNA to ensure the collection of data including repetitive regions and their adjacent non-repetitive loci. This demonstrates that repeat content is not a fundamental limitation of dense optical genome assembly; rather, the critical factor is the ability to detect, classify, and appropriately weight repetitive and long-range signal features during assembly.

Another key insight of this study is that long-range intensity trends, spanning hundreds of kilobases, contribute substantially to barcode similarity across distinct loci. In *S. pombe*, these trends manifest as chromosome-end GC gradients and centromeric low-GC domains, which are consistently detectable in both theoretical and experimental barcodes. Unlike sparse motif-based OGM, which samples genomes only at discrete labeling sites, dense CB-based OGM captures continuous sequence-dependent fluorescence intensity along the entire DNA molecule. This enables the detection of extended compositional trends and repetitive regions with low motif density that are not directly accessible through sparse labeling strategies. Since correlation-based similarity metrics evaluate agreement across a defined window, shared low-frequency compositional trends can contribute substantially to the overall similarity score, partially masking differences in local (5–20 kbp) sequence-dependent features. Consequently, unrelated genomic regions exhibiting similar long-range intensity trends may appear more similar than expected based on their local barcode features alone. While such trends can bias correlation-based metrics and promote spurious alignments, they also reflect large-scale chromosomal sequence organization that is inaccessible to sparse-label OGM approaches. Accounting for this signal component will therefore be essential for future OGM assembly frameworks.

Together, these findings position dense OGM as both an assembly tool and a probe of large-scale genomic sequence organization. While further methodological refinement will be required to fully exploit long-range signal information in even more complex genomes, the chromosome-scale yeast assemblies presented here establish an important foundation for extending dense OGM to larger eukaryotic systems and to biologically and clinically relevant repetitive loci.

From a methodological perspective, the combination of repeat-aware masking and characterization of long-range intensity profiles provides a generalizable strategy for extending dense, CB-based, OGM beyond yeast. Many of the challenges encountered here, such as repetitive arrays and long-range compositional structure, are even more common in mammalian genomes, where repetitive regions constitute over half of the human genome and remain systematically underrepresented or collapsed in many reference assemblies^32,33^. Despite recent advances, such as telomere-to-telomere assemblies, large repetitive loci remain difficult to characterize robustly with sequencing alone, particularly when copy number, structural heterogeneity, or long-range organization are biologically relevant^32^. In this context, dense OGM offers a complementary view by capturing continuous intensity profiles over hundreds of kilobases, preserving long-range compositional patterns and repetitive genomic features that can be difficult to characterize using sequencing alone. Since these genomic features are substantially more prevalent in eukaryotic genomes than in bacterial genomes, we anticipate that similar considerations will become increasingly important for dense OGM analyses beyond yeast genomes. While evaluation in human genomes lies beyond the scope of this study, the framework presented here suggests a path toward resolving medically relevant repeat structures, including large satellite arrays, rDNA clusters, and disease-associated macrosatellites^3,18^.

An additional advantage of CB-based, dense OGM is that the intensity variation signal arises from YOYO-1 labeling of DNA, a fluorophore widely used in many OGM and single-molecule assays for DNA visualization. Therefore, the incorporation of CB-based staining does not require additional excitation sources, emission filters, or major instrumentation changes, making this signal layer effectively available in existing platforms. This creates opportunities to combine CB-based OGM with other labeling strategies, such as enzymatic motif labeling, damage mapping^34^, or epigenetic assays^15^, while retaining long-range genomic context. In this way, CB-based OGM is not limited to genomic reconstruction, but can serve as a scaffold for integrating multiple molecular readouts along individual DNA molecules.

In conclusion, this study demonstrates that dense, continuous optical genome mapping can be extended from bacterial to eukaryotic genomes when eukaryotic structural constraints are appropriately considered. By combining repeat-aware masking, correlation-based bargroup assembly, and reference-assisted refinement of unresolved joins, DOGMA reconstructs chromosome-scale optical maps in yeast without collapsing non-adjacent loci. Importantly, CB-based OGM preserves large-scale compositional features of chromosomes, enabling analysis of genomic structure beyond strictly local sequence similarity. Together, these results establish DOGMA as a robust framework for eukaryotic optical genome assembly and lay the groundwork for applying dense OGM to increasingly complex genomes.

## Methods

### Yeast cultivation and DNA extraction

Experiments were performed using *Saccharomyces cerevisiae* strain BY4742 and *Schizosaccharomyces pombe* strain NS112 (*leu1-32 his3-D1 ura4-D18 ade6-M210*). The protocol for DNA extraction was adapted from previous work^35^. In short, *S. cerevisiae* was grown overnight and used to inoculate a culture that was grown at 30 °C for 6 to 8 h to reach an OD600 of 0.1. Afterwards, 6 × 10^8^ cells were harvested and frozen at −20 °C until the DNA extraction was performed. For *S. pombe*^34^, 50 mL exponentially growing cells at initial concentration of 0.5 million cells/mL were grown in Edinburgh minimal media for 16 h at 30 °C until they reached 14 million cells/mL. Cells were harvested by centrifugation at 1500 × *g* for 5 min and washed twice with 50 mM cold EDTA. Cells were frozen at −20 °C prior to DNA extraction. The extraction of DNA from both types of yeast cells was performed using the CHEF Yeast Genomic DNA Plug Kit from BIO-RAD. In short, 1 mL of agarose plugs were created for each strain by combining 6 × 10^8^ cells with 625 µL CSB (Cell Suspension Buffer) and 375 µL 2% CCA (CleanCut agarose). Agarose plugs were then treated with Lyticase, RNase, and Proteinase K, and washed several times according to the CHEF Yeast Genomic Plug Kit protocol.

DNA was then extracted from the agarose plugs. This is done by first incubating in 1x CutSmart (New England Biolabs) solution at 70 °C for 10 min, followed by incubation at 42 °C for 10 min. This was followed by a 42 °C incubation with 2 U agarase for 2 h (New England Biolabs). DNA was then quantified by using Qubit dsDNA Broad Range Assay kit (Invitrogen).

### CB-based OGM lab procedures

Densely labeled DNA molecules were obtained by a single-step competitive binding (CB) based staining reaction, as described previously^35^. In short, the CB staining reactions were performed by adding 4–6 µM DNA (in bases), 0.4–0.6 µM YOYO-1, and 120–180 µM netropsin in a 10:1:300 ratio to a 0.5X TBE (Tris-Borate EDTA) 10 µL solution. DNA from bacteriophage λ (48,502 bp bought from Roche) was added to make up ~30% of the DNA mass in the staining reactions, to serve as an internal size reference. Finally, an incubation at 50 °C for 30 min was followed by a 15-fold dilution with MQ-water and 3 µL of β-mercaptoethanol (BME) to reduce photodamage.

### Nanofluidic experiments

Stained DNA molecules were stretched for imaging using a nanofluidic device whose fabrication is described elsewhere^7,36,37^. These devices are designed to have two sets of two loading wells, where each set is connected via a separate microchannel. Both microchannels connecting the loading wells are connected to each other by 120 parallel nanochannels, each having a cross section of 100 × 150 nm^2^ and extending 500 µm in length. To ensure uniform conditions, the chips underwent pre-conditioning with 0.033X TBE buffer supplemented with 2% BME v/v prior to sample loading. DNA loading was done by adding 10 µL of stained sample into one of the loading wells, while the others contained the same buffer used for pre-conditioning, but no DNA. DNA molecules were then driven first into the microchannels and then the nanochannels by pressure-driven N_2_ flow. Stretched and stained DNA molecules in the nanochannels were then imaged by using an inverted fluorescence microscope (Zeiss Axio Observer D.1) equipped with a 63x oil immersion objective (NA = 1.46, Zeiss), a Colibri 7 light source (Zeiss), and a Photometrics Evolve EMCCD camera. Systematic acquisition of videos of up to 100 frames was carried out for each molecule, utilizing an exposure time of 100 ms and a FITC filter set (Zeiss).

### Barcodes processing

Barcodes were generated from videos using a previously developed custom MATLAB software called lldev’^38,39^. In short, DNA molecules are detected in each time frame within a movie. The detected DNA molecules are then turned into kymographs, where each row of pixels represents the intensity along the DNA molecule in a single frame. The kymographs are aligned to compensate for small thermal fluctuations of the DNA inside the nanochannels. Aligned kymographs are then processed by a custom quality control (QC) algorithm to merge molecules spanning multiple fields of view (multi-FoV) and to remove molecules that show a significant change in size during imaging (see Supplementary Methods 1). Finally, the filtered and aligned kymographs are time-averaged, rendering a time series-like 1D intensity trace along the extension of the DNA molecule, referred to as a barcode.

### Reference alignment

Reference genome sequences were used as an input to the HCA package^12^ to in silico generate theoretical reference CB-based barcodes that can be used for alignment and coverage analysis. Default HCA-theory settings were used to create said reference barcodes except for selecting for linear DNA, 0.25 nm/bp for DNA extension, 254 nm/px for pixel width, and a psfSigmaWidth of 681.81 nm. For *S. cerevisiae*, the S288C reference genome (R64 assembly), obtained from Saccharomyces Genome Database^27^, was used as reference since the BY4742 strain was derived from S288C. For *S. pombe*, the reference genome of strain 972 h − , obtained from PomBase^28^, was used as a reference since strain NS112 (*leu1-32 his3-D1 ura4-D18 ade6-M210*) was derived from the 972 strain background.

Genome-wide barcode alignment was performed with a full-length similarity search alignment strategy to estimate coverage across the reference genomes. This approach, contained within the HCA package and implemented in previous work^35^, is based on a Pearson correlation coefficient (CC) scoring system to identify the best placement for a query barcode within a given reference. Default HCA-alignment settings were used for barcode alignment except for a 0.01 step stretch and the use of both local and global alignment options. The difference between the CC score of the highest match and second-highest match is used along this paper as ∆CC. This parameter is used as an indicator of alignment ambiguity, where low ∆CC scores represent lower certainty of alignment, or higher ambiguity. In this strategy, the full query barcode is aligned to a segment of the reference, and therefore requires the query to be shorter than the reference.

To identify individual molecules spanning complete *S. cerevisiae* chromosomes, a local similarity alignment was used in a barcode-to-reference mode. This approach uses a sliding window to identify the highest-scoring subregions between query and reference, allowing partial matches and overhang beyond reference boundaries. Both query and reference must exceed the selected overlap window length. The local alignment is based on a matrix profile framework and is also used in a barcode-to-barcode mode as the core function for pairwise similarity calculations in DOGMA^14,40^. For each chromosome, an overlap window corresponding to 70% of the chromosome length was used. Candidate barcodes were restricted to lengths between 70% and 150% of the corresponding chromosome size to accommodate molecule length variability while ensuring chromosome-scale coverage.

Following the initial de novo bargroup assembly, bargroups were aligned to the reference genomes using local similarity alignment rather than full-length alignment. This choice accounts for assembled contigs that may extend over the edges or exceed the full length of the corresponding reference chromosomes due to the presence of insertions, repetitive or unresolved genomic segments. The resulting reference placements were used to identify unresolved joins between bargroups. Bargroups with consistent reference placements were subsequently merged using local bargroup-to-bargroup similarity to generate chromosome-scale assemblies. Local alignments were calculated using an overlap window corresponding to 70% of the non-repetitive portion of each contig. We refer to this post-assembly use of reference placement to identify and resolve remaining joins as reference-assisted refinement.

For reference-based analysis of the assembled bargroups, chromosome XII of the *S. cerevisiae* genome was split into left and right side after noticing partial alignment due to repetitive regions not included in the reference. The breakpoint for the insertion of repetitive region into chromosome XII was manually determined to be around position 472 kbp.

### Repetitive finder

Since DOGMA uses local barcode-to-barcode alignment for its bargrouping procedure, repetitive regions shared between molecules can dominate the matching process, potentially leading to non-canonical groupings independent of the non-repetitive content elsewhere in the molecules. For this reason, it is important to identify molecules with repetitive regions and mask the repetitive part of them so that the rest of the molecule can be used as a contextual “anchor” in the bargrouping process. This way, bargroups are created based on the similarity of the non-repetitive parts of each barcode, while the repetitive parts are still preserved and can be “unmasked” for downstream analysis.

To identify and mask highly repetitive regions prior to assembly, we developed a custom *Repetitive Finder* algorithm implemented in MATLAB (Supplementary Fig. 2). The method detects periodic patterns in barcode intensity profiles using the autocorrelation function to estimate repeat periodicity, followed by Fourier transform validation. A rolling-window correlation then identifies continuous repetitive regions, with segmentation achieved using the findchangepts function (Supplementary Methods 2). Regions with an average correlation >0.4 across at least five repeat units are marked as repetitive and masked in subsequent assembly steps. These empirically determined thresholds effectively separate highly repetitive barcodes from non-repetitive ones (Supplementary Fig. 2).

### DOGMA pipeline implementation and bargroup quality metrics

Assembly was performed using the previously developed^14^ DOGMA algorithm. In short, pairwise barcode comparisons were evaluated using Pearson correlation coefficient under constrained alignment parameters, including stretch tolerance, positional tolerance, and statistical significance thresholds defined by the DOGMA null model framework (Supplementary Table 1).

Because repetitive, partially repetitive, and non-repetitive barcode classes exhibit distinct similarity distributions, parameter values within the null-model-based *p*-value framework were optimized separately for each class using the parametrization.m script within the DOGMA package (Supplementary Table 2). This parametrization is recommended to be run once per experimental setup, since parameters are greatly affected by pixel size and effective PSF of the set up.

To enable incorporation of repetitive regions, assembly was performed in multiple stages reflecting barcode class. Independent DOGMA assemblies were first carried out on non-repetitive and partially repetitive masked barcodes. Repetitive barcodes were then assembled in a subsequent stage using the partially repetitive assemblies as anchoring context. This staged strategy prevents repetitive segments from driving initial grouping while allowing their incorporation once non-repetitive context has been established. Finally, all resulting bargroup contigs were merged through an additional DOGMA-based local assembly step to generate unified bargroups spanning all barcode classes. Consensus bargroups were constructed by averaging aligned barcode intensity profiles following stretch normalization. To mitigate grouping driven by long-range low-frequency trends and to ensure that only structurally consistent and information-rich groups were retained, three complementary quality metrics were evaluated for each bargroup.

Internal similarity was first assessed using pairwise Pearson cross-correlation coefficients (CC) computed between all barcodes within each bargroup and their consensus. The mean of these values (meanCC) was required to exceed 0.7, ensuring overall coherence among members. The standard deviation of the CC scores (stdCC) was also evaluated to measure internal consistency; bargroups with stdCC > 0.05 were discarded, as high dispersion indicates heterogeneous membership despite acceptable mean similarity.

Finally, a signal-to-noise ratio (SNR) was computed to quantify the strength of the consensus structure relative to variability among its members. For a bargroup with N members, let ${B}_{i}(x)$ denote the intensity at pixel x of barcode i, and ${\bar{B}}(x)=\frac{1}{N}{\sum }_{i=1}^{N}{B}_{i}\left(x\right)$ the consensus intensity. The SNR was defined as in Eq. (1) where the numerator reflects variation across the consensus profile, and the denominator reflects the average per-pixel variability among individual barcodes. Bargroups with SNR < 1.0 were excluded.

1 $${{{\rm{SNR}}}}=\frac{{{{{\rm{std}}}}}_{x}[\bar{B}(x)]}{{{{{\rm{mean}}}}}_{x}[{{{{\rm{std}}}}}_{i}[{B}_{i}(x)]]}$$

Only bargroups satisfying all three criteria were retained for downstream assembly.

Barcodes that remained unassembled but exhibited high-confidence reference alignments and low alignment ambiguity (∆CC > 0.03) were retained for downstream reference-based analysis and used to investigate and, when necessary, restore local contiguity breaks, as described in the results.

### Statistics and reproducibility

The assembly process requires enough DNA molecules to be captured to cover the whole genome several times. For this reason, the amount of molecules collected was continuously evaluated by estimating genome coverage as the ratio of total collected DNA and total genome length. Collection continued until each genome was covered at least 10x. For *S. pombe* additional molecules were collected to account for the extended chromosome length. DNA molecules shorter than 150 kbp, and not passing our QC-filtering (Supplementary Information) were excluded from analysis. The investigators were not blinded to allocation during experiments and outcome assessment.

### Reporting summary

Further information on research design is available in the Nature Portfolio Reporting Summary linked to this article.

## Supplementary information

Supplementary Information file

Reporting summary

Transparent Peer Review file

## Source data

Source Data

## Data Availability

The data generated and analyzed during this study is available in Zenodo with the identifier https://doi.org/10.5281/zenodo.21507295^41^. Raw microscopy images, preprocessed barcodes extracted from the images, processed barcode data and assembly outputs in the form of.mat files, are included in these deposits. Genome sequences used in this work include: *S. cerevisiae S288C R64* GenBank accession number GCA_000146045.2 [https://www.ncbi.nlm.nih.gov/datasets/genome/GCA_000146045.2/], *S. pombe 972h-* under the GenBank accession number GCA_000002945.3 [https://www.ncbi.nlm.nih.gov/datasets/genome/GCA_000002945.3/], and *S. cerevisiae S288C* long read assembly under GenBank accession number GCA_002057635.1 [https://www.ncbi.nlm.nih.gov/datasets/genome/GCA_002057635.1/]. Source data are provided with this paper.

## Code availability

The custom MATLAB code used for barcode processing and assembly are available in GitHub (https://github.com/dnadevcode/yeast_denovoassembly) and through Zenodo with the identifier https://doi.org/10.5281/zenodo.21507295^41^.
