# Supplementary material for: Chromosome-scale assembly of yeast genomes using enzyme-free, dense optical genome mapping
Source: Nat Commun. 2026 Oct 1;17:10404. doi: 10.1038/s41467-026-78211-7 (PMC13631253; doi:10.1038/s41467-026-78211-7)
Supplement: Supplementary file 1 [file 41467_2026_78211_MOESM1_ESM.pdf]

Chromosome-scale assembly of yeast genomes using enzyme-free, dense optical genome mapping

Supplementary Information

Luis Mario Leal-Garza[1], Hanna Zachrisson[1], Albertas Dvirnas[1,2], Gaurav Goyal[1], Ikenna Obi[3], Sriram KK[1], Nasim Sabouri[3,4], Tobias Ambjörnsson[5] and Fredrik Westerlund[1*]

[1]Department of Life Sciences, Chalmers University of Technology, Gothenburg, Sweden,

[2]Department of Mathematics and Mathematical Statistics, Umeå University, Umeå, Sweden.

[3]Department of Medical Biochemistry and Biophysics, Umeå University, Umeå, Sweden

[4]Science for Life Laboratory, Umeå University, Umeå, Sweden

[5]Department of Earth and Environmental Sciences, Lund University, Lund, Sweden

[*]Corresponding author: fredrikw@chalmers.se

Supplementary Methods

Supplementary Methods 1. Quality control of barcode extraction

To ensure accurate barcode generation, quality control procedures were applied to remove artifacts arising from DNA dynamics within nanochannels and from imaging constraints.

Due to the lack of complete immobilization in nanochannels, DNA molecules require a relaxation period to reach their equilibrium extension. This relaxation time scales with the square of the DNA contour length[1], causing longer molecules to exhibit a transient "shrinking" behavior prior to stabilization. Barcodes extracted from such non-equilibrated kymographs may contain distorted or overlapping features, compromising downstream analysis.

To address this, a shrink-filtering algorithm was applied prior to kymograph alignment to identify and exclude molecules that had not reached equilibrium. A detailed description of this procedure is provided in previous studies [2].

Following shrink filtering, kymographs were processed using a custom QC pipeline to identify and merge molecules spanning multiple fields of view (multi-FoV) (Supplementary Fig. 1). Candidate barcode pairs were identified based on acquisition metadata and subsequently aligned using local barcode-to-barcode alignment. Overlapping regions of at least 130 pixels near the molecule ends were required. Merged barcodes were generated by averaging normalized intensity profiles across the aligned region. This approach enables reconstruction of molecules exceeding the imaging field of view (~500 kbp), improving representation of long DNA fragments. Since overlapping barcode segments are merged into a single reconstructed molecule rather than concatenated end-to-end, this procedure only has a minor effect on the overall molecule-length distribution.

Supplementary Methods 2. Detection and masking of repetitive barcode regions

To reduce the impact of repetitive sequence content on alignment and assembly, repetitive regions within barcodes were identified and masked prior to downstream analysis. The presence and location of repetitive regions within barcodes was identified using a custom algorithm based on autocorrelation for periodicity analysis, frequency domain validation and local similarity for segmentation. All the algorithms mentioned below were performed using default parameters for all the functions unless explicitly stated and executed using MATLAB R2023b Update 7 (23.2.0.2515942).

For each barcode, the autocorrelation function (ACF) was calculated using `autocorr` (Supplementary Fig. 2x.i). Sinusoidal curves in ACF emerge when the barcode is either fully periodic or partially periodic, but non periodic regions in a barcode can hinder the interpretation of ACF. To facilitate this, only the first 110 lags of the ACF are analyzed, and the ACF is detrended using `detrend`. Peaks in ACF were then detected using `findpeaks` and the repeat unit length was estimated as the mean distance between the first six peaks. Periodicity was considered

significant only if the spacing between them was consistent, quantified as a coefficient of variation lower than 20%. Barcodes failing this criterion were classified as non-periodic. If, on the other hand, a consistent repeat unit length is estimated, the barcode is flagged as repetitive and proceeds to the rest of the analysis.

The repeat unit length estimate is then validated in the frequency domain using a Fast Fourier Transform (FFT) (Supplementary Fig. 2x.ii). To do this, FFT is calculated using `fft` and peaks representing the possible repetitive unit's frequency are detected using `findpeaks` (MinPeakProminence=5). The frequency with the maximum amplitude detected in the FFT is then compared to the length of the repetitive unit calculated from the ACF to determine if the repetition's size is consistent. FFT-estimated repeat unit length should be within 10% of the value of ACF-estimated repeat unit length. Failure of this consistency check would render barcodes as non-repetitive.

For barcodes passing this consistency check, the location of repetitive regions was determined using a rolling-window correlation analysis (Supplementary Fig. 2x.iii). A window size corresponding to the estimated repeat unit was used to compute correlations between adjacent segments along the barcode, yielding a position-dependent similarity profile. The `findchangepts` function was then used on the rolling-window correlation (MaxNumChanges=2) to try to find segments of the molecule that have higher self-correlation than others. To facilitate interpretation, we limit the sensitivity of repetitive region detection to three sections for each barcode, each of which can be repetitive or non-repetitive. This limitation was chosen since at most, we have observed that barcodes can contain up to two non-repetitive regions separated by a repetitive one.

Each segment was classified as repetitive or non-repetitive based on its mean correlation value, using a threshold of 0.4. Contiguous segments exceeding both this threshold and a minimum length of five repeat units were defined as repetitive regions. Barcodes were further categorized as fully repetitive if non-repetitive regions were shorter than 150 pixels, corresponding to the overlap window size used during pairwise barcode comparison in the assembly process; otherwise, they were classified as partially repetitive (Supplementary Fig. 2x.iv).

The output of this algorithm provides a categorical flag indicating non-repetitive, partially repetitive, or fully repetitive barcodes (rep_check = 0, 1, or 2 respectively), and a positional mask identifying repetitive regions within each barcode (rep_mask). These masks were used to exclude repetitive regions during alignment and assembly while preserving their positions for downstream analysis. All thresholds were empirically selected to balance sensitivity to periodic structure with robustness to noise and barcode length variability.

Supplementary Figures

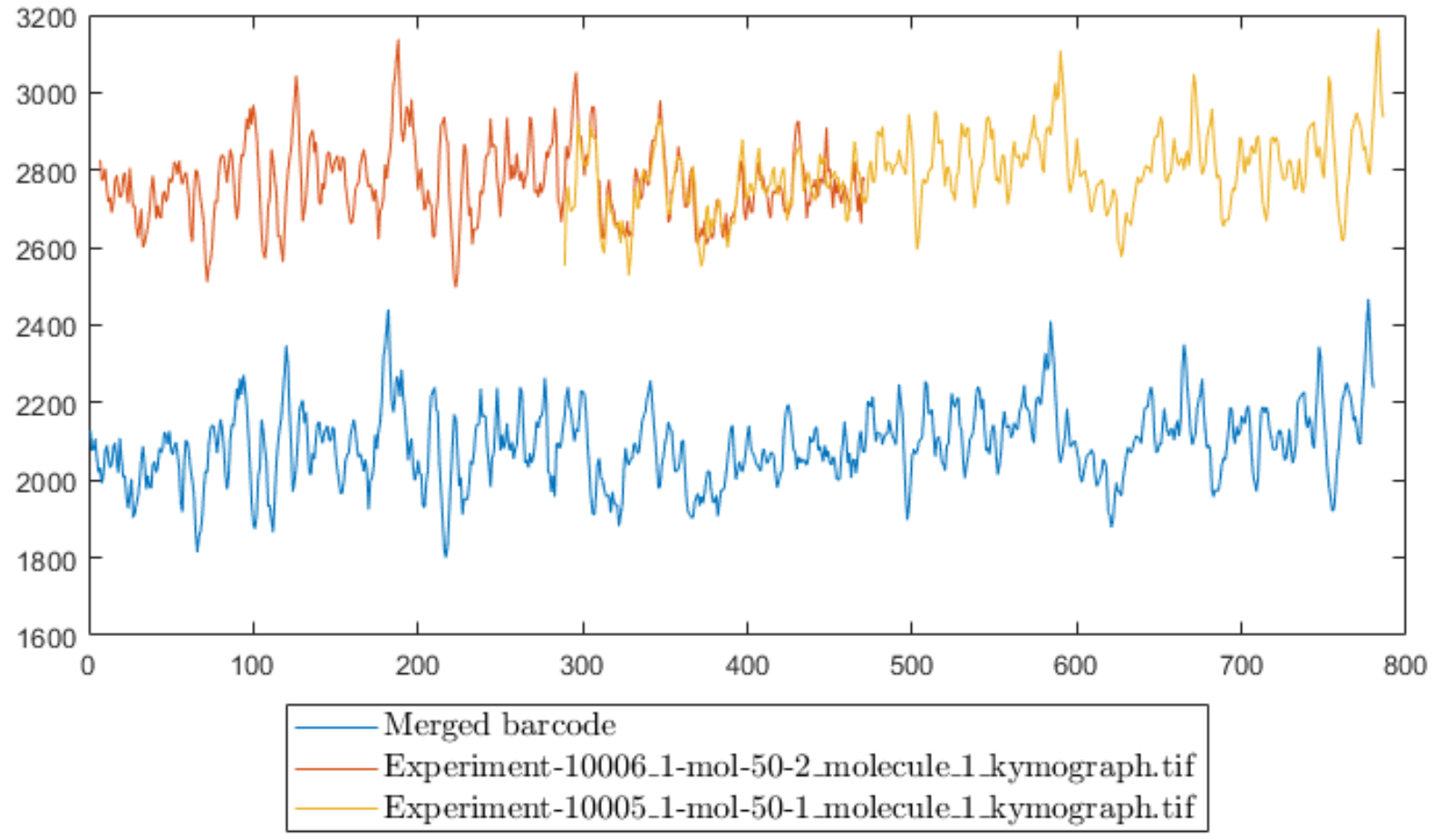

Supplementary Fig. 1 | Multi-FoV barcode merging. Two experimental barcodes (red and yellow) derived from imaging of a single 792 kbp DNA molecule across overlapping fields of view. Local alignment of the overlapping regions enables merging into a single consensus barcode (blue), which is used for downstream analysis. Source data are provided as a Source Data file.

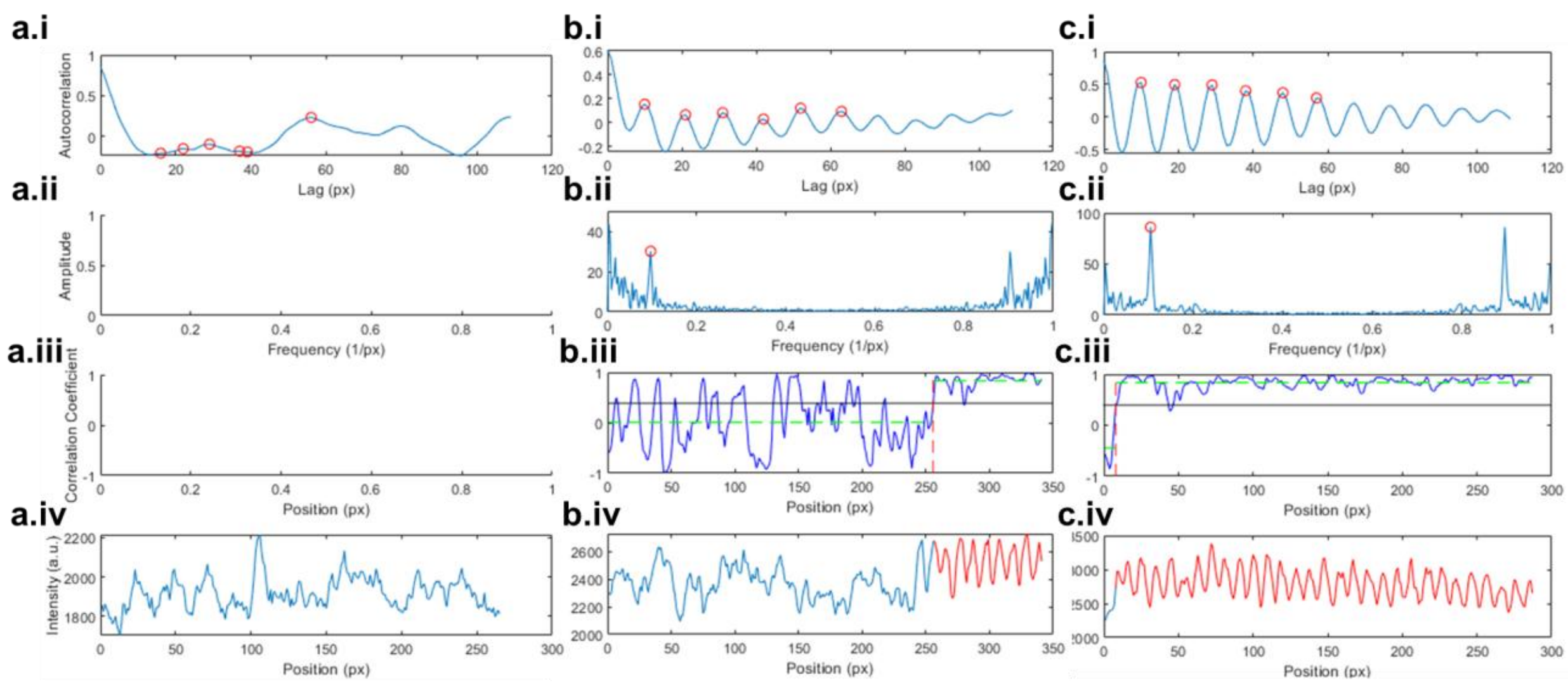

Supplementary Fig. 2 | Repetitive Finder. a A non-repetitive, **b** a partially repetitive and **c** a fully repetitive molecule are shown to illustrate the workflow. **i** Autocorrelation analysis used to detect periodic signal patterns and estimate repeat size. Process is halted and molecule regarded as non-repetitive if no periodic signal is detected here. **ii** Validation of repeat periodicity using FFT. **iii** Rolling-window correlation and segmentation using change-point detection (`findchangepts`) function. **iv** Final detection and classification of repetitive (red) and non-repetitive (blue) regions in barcodes. Source Data and Source Code underlying the plots in this figure are available on Zenodo.

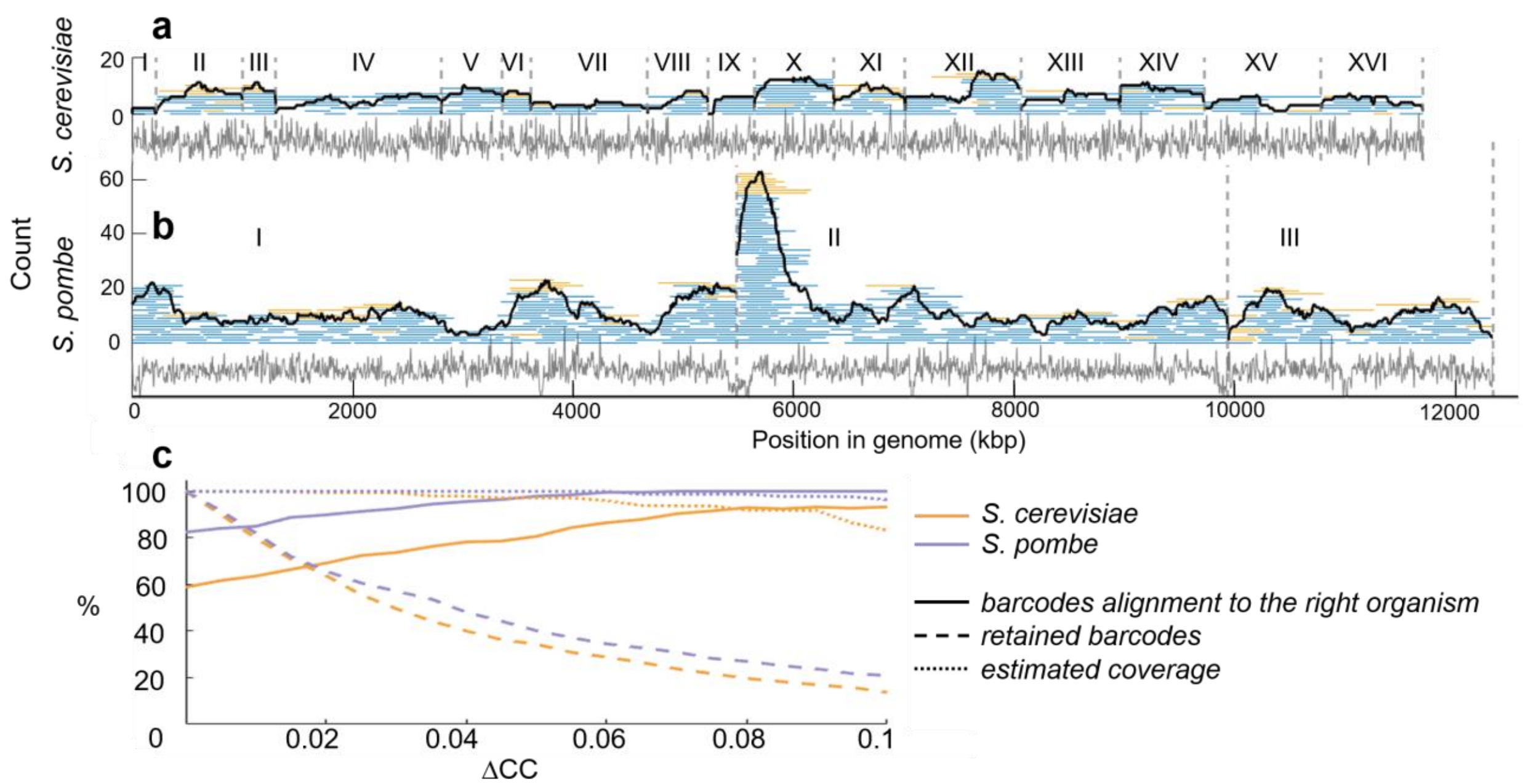

Supplementary Fig. 3 | Alignment of barcodes passing QC to reference genomes and ΔCC filtering. Alignment of barcodes passing a QC and ΔCC (0.03) filter to reference genomes of **a** *S. cerevisiae* and **b** *S. pombe*. Correct species alignments are shown in blue and incorrect alignments in yellow. Black lines indicate coverage depth. **c** Percentage of barcodes aligning to the correct organism (solid lines), percentage of retained barcodes (dashed lines) and percentage of estimated coverage (dotted lines) after using a local barcode-to-reference alignment of repeat-masked barcodes under varying thresholds of ΔCC (difference in CC-score between the first- and second-best match for each molecule) for *S. cerevisiae* (orange) and *S. pombe* (purple). Source Data and Source Code underlying the plots in this figure are available on Zenodo.

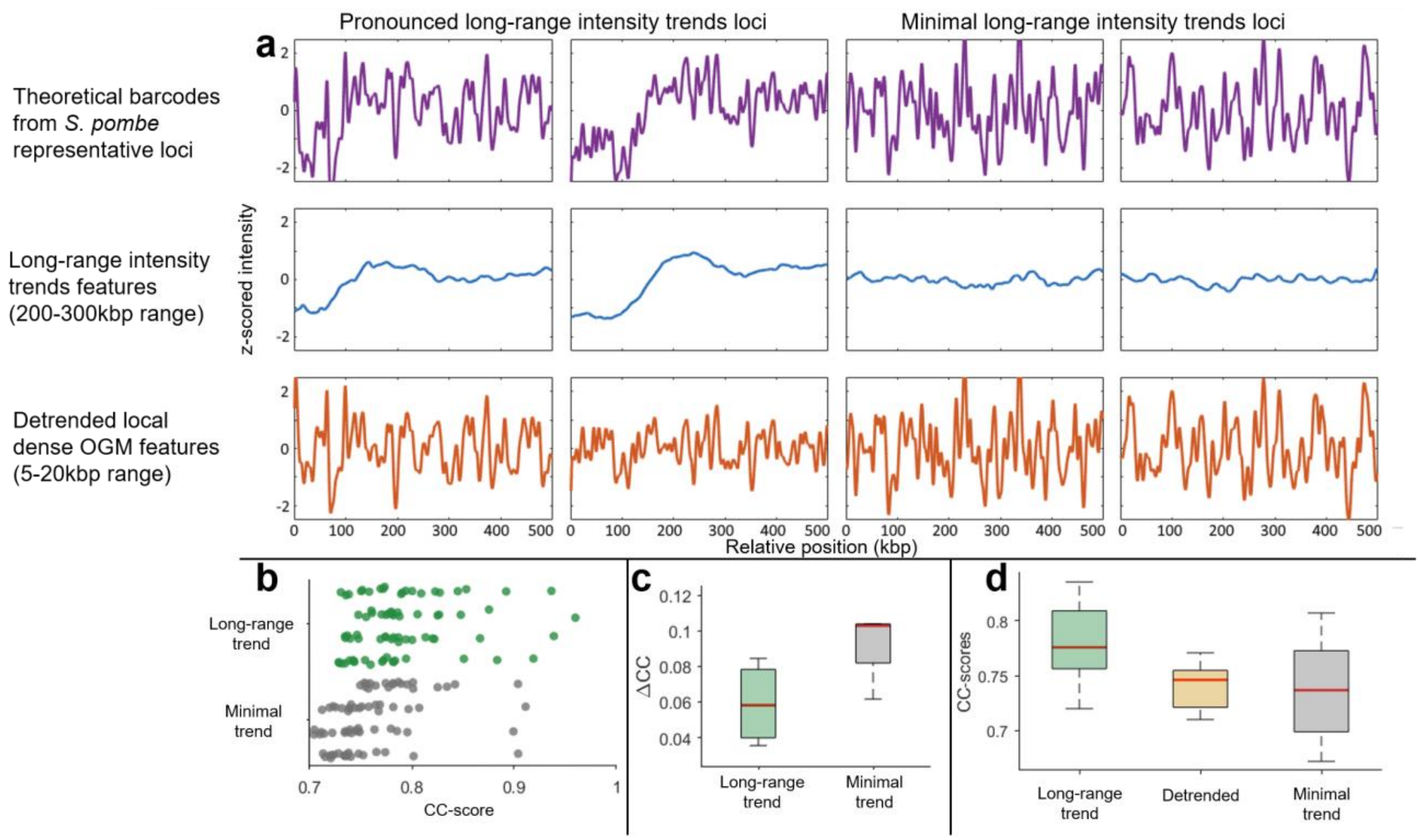

Supplementary Fig. 4 | Long-range intensity trends increase correlation-based similarity and contribute to alignment ambiguity. a Representative theoretical barcodes (purple, top) from different regions of the *S. pombe* reference genome exhibiting either pronounced or minimal long-range intensity trends. For visualization, each barcode is decomposed into a low-frequency component (blue, middle) and a detrended component highlighting local features (orange, bottom). **b** Top alignment scores obtained by aligning eight synthetic experimental barcodes (generated by adding experimental noise to the theoretical barcodes) against the *S. pombe* reference. Barcodes containing pronounced long-range intensity trends (green) produce higher scores, not only at their correct loci (rightmost) but also at unrelated loci, increasing alignment ambiguity. **c** Distribution of ΔCC (difference between the best and second-best alignment scores) showing reduced ΔCC for barcodes with pronounced long-range intensity trends (green). **d** Distribution of pairwise correlation scores between simulated barcodes. Pronounced long-range intensity trends increase correlation between unrelated barcodes (green), whereas detrending (orange) restores the correlation distribution to that of barcodes with minimal long-range intensity trends (gray) Source Data and Source Code underlying panel **a** in this figure are available on Zenodo. Source data for panels b-d are provided as a Source Data file.

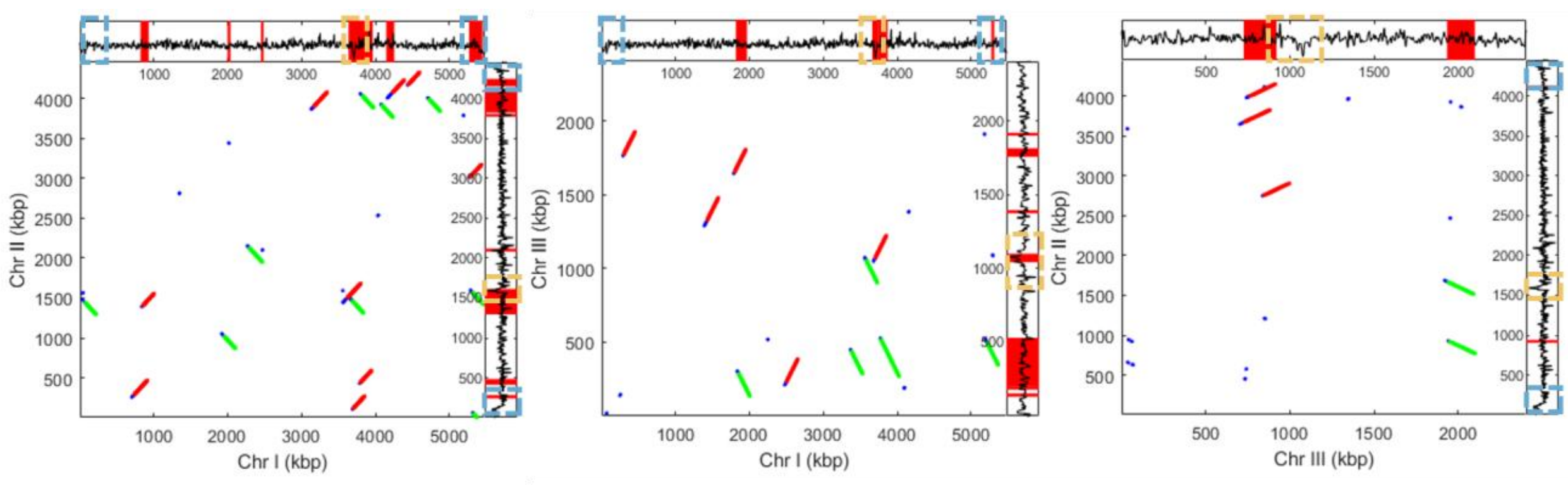

Supplementary Fig. 5 | Intragenomic self-similarity analysis. Alignment start points (blue) were identified for segments ≥150 kbp with CC ≥ 0.75. Regions with ≥10 consecutive matches were extended by 150 kbp and classified as forward (red) or reverse (green) matches. Regions with multiple overlapping matches are indicated as similarity hotspots. Genomic regions exhibiting pronounced long-range intensity trends in Fig.3c are indicated by the corresponding blue and yellow dashed boxes to facilitate comparison between the two analyses. Source Data and Source Code underlying the plots in this figure are available on Zenodo.

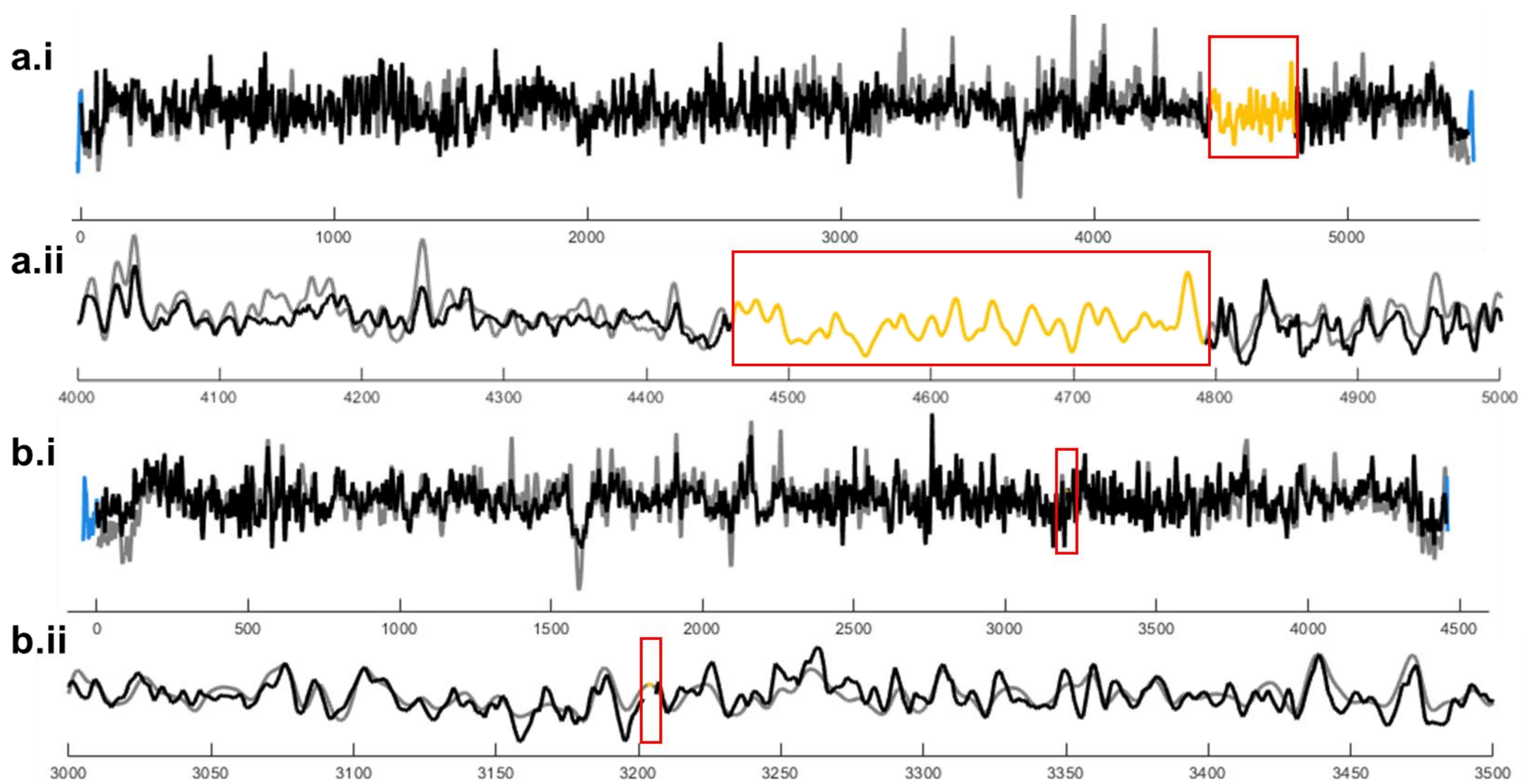

Supplementary Fig. 6 | Contiguity gaps in assembled genomes. Gaps in contiguity (yellow) observed after aligning assembled bargroups (black) to reference barcodes (gray) **a** for chromosome I and **b** chromosome II of *S. pombe*. Full chromosome views are shown in **a.i**, **b.i**, with corresponding zoomed-in regions of 1 Mbp and 0.5 Mbp in **a.ii**, **b.ii**, respectively, highlighting representative gaps (red boxes). Bargroup segments extending beyond reference boundaries are shown in blue. Source Data and Source Code underlying the plots in this figure are available on Zenodo.

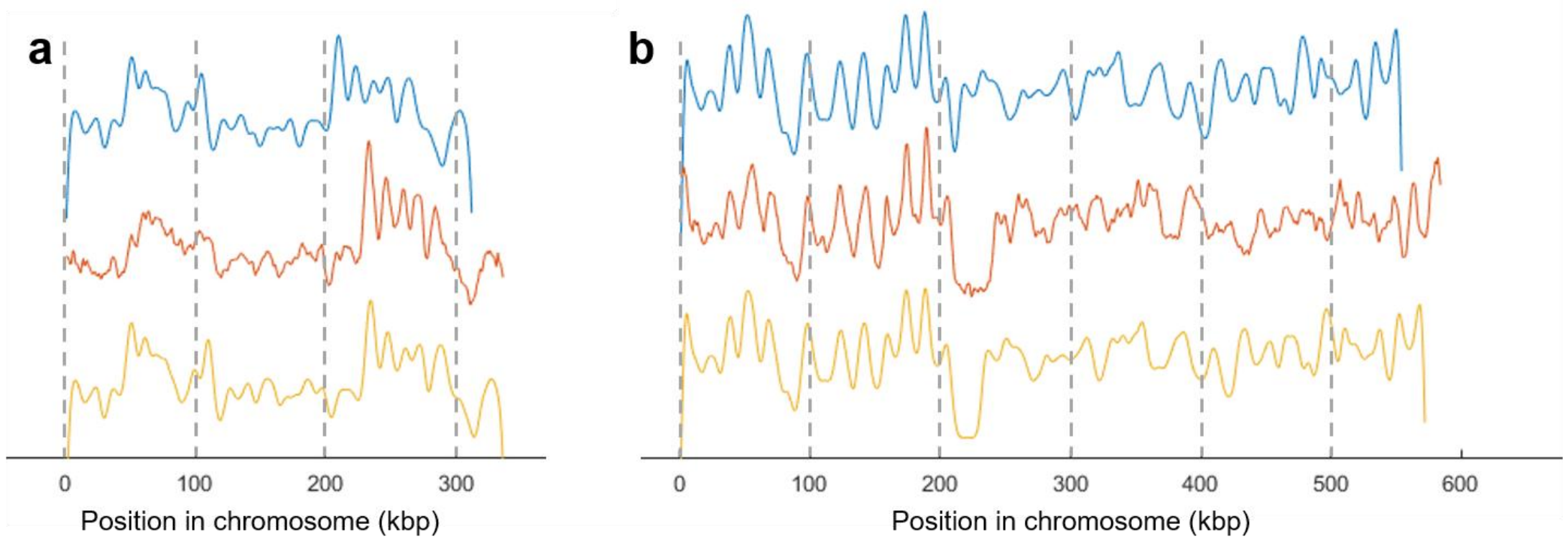

Supplementary Fig. 7 | Reference assembly discrepancies. Regions of discrepancy were observed between the assemblies (red) and theoretical barcodes generated from the S288C reference genome (blue) of **a** chromosome III and **b** chromosome VIII of *S. cerevisiae*. Theoretical barcodes generated from a long-read assembly (yellow) show improved agreement to our assemblies compared to the R64 reference genome. Source data are provided as a Source Data file.

Supplementary Tables

Supplementary Table 1 | Parameters used for DOGMA assembly, including stretch tolerance, positional tolerance, overlap window size, and statistical thresholds.

Variable	Description	Value
w	overlap window	150px
sF_{step}	length re-scale step	0.01
sFmax	max re-scaling factor	0.15
scDiffSetting	allowed scale factor difference	0.05
pxDiffSetting	allowed pixel difference	10px
p_{thresh}^{local}	p-value local threshold	0.05
$p_{thresh}^{leftover}$	p-value leftover threshold	0.05

Supplementary Table 2 | Null-model parameters used for bargroup formation across barcode classes (non-repetitive, partially repetitive, and fully repetitive). Values are fitted using the `parametrization.m`[2] script which uses the experimental data as input.

Variable	Non-repetitive	Semi-repetitive	Full-repetitive
α_v	0.1749	0.1689	0.0533
α_N	0.1461	0.1618	0.0734
β_v	0.2415	0.1773	0.0200
β_N	0.0144	0.0254	0.0371

Supplementary Table 3 | Quality metrics (meanCC, stdCC, SNR) for bargroups included in the final assemblies.

	Chromosome	meanCC	stdCC	SNR
S. cerevisiae	I	0.8828	0.00036	1.8224
	II	0.7593	0.01704	1.2200
	III	0.8034	0.00337	1.3002
	IV	0.9262	0.00131	1.8507
	V	0.7980	0.00267	1.2607
	VI	0.8278	0.00095	1.3956
	VII	0.7847	0.00498	1.0568
	VIII	0.7476	0.00449	1.0435
	IX	0.7788	0.00382	1.1912
	X	0.7980	0.00073	1.2475
	XI	0.8836	0.00056	1.5581
	XII (left part)	0.9189	0.00002	1.7716
	XII (right part)	0.8309	0.00667	1.4978
	XIII	0.8336	0.00758	1.4576
	XIV	0.7795	0.00357	1.1589
	XV	0.8977	0.00380	1.2828
	XVI	0.8668	0.00509	1.3542
S. pombe	I	0.8033	0.01025	1.3347
	II	0.8078	0.00427	1.2904
	III	0.8297	0.00499	1.3777

Supplementary Table 4 | Quality metrics for the assemblies before and after the strategies presented in this work were introduced. For each chromosome, the number of bargroups resulting from assembly that aligned to it (N) are shown together with their total length, assuming correct alignment. Coverage is shown as a percentage of the reference length. Mean local CC shows the mean of the local alignment scores of the bargroups aligned to each chromosome.

			Assembly before introduced strategies				Assembly after introduced strategies			
Organism	Chromosome	Reference Length (kbp)	N	Inferred Length (kbp)	Coverage (%)	Mean local CC	N	Inferred Length (kbp)	Coverage (%)	Mean local CC
S. cerevisiae	I	215	1	236	109.77	0.81	1	228	106.05	0.91
	II	788	3	829	105.2	0.67	1	800	101.52	0.79
	III	300	1	684	228	0.84	1	336	112	0.93
	IV	1496	1	440	29.41	0.67	1	1486	99.33	0.79
	V	556	0	0	0	0	1	575	103.42	0.93
	VI	254	1	261	102.76	0.7	1	265	104.33	0.93
	VII	1062	0	0	0	0	1	1077	101.41	0.82
	VIII	542	1	585	107.93	0.87	1	584	107.75	0.95
	IX	421	1	419	99.52	0.79	1	432	102.61	0.92
	X	722	0	0	0	0	1	740	102.49	0.95
	XI	644	0	0	0	0	1	654	101.55	0.94
	XII	1049	1	514	49	0.84	2	1496	142.61	0.87
	XIII	898	0	0	0	0	1	902	100.45	0.84
	XIV	760	1	711	93.55	0.79	1	767	100.92	0.91
	XV	1062	1	685	64.5	0.72	1	1050	98.87	0.83
	XVI	921	0	0	0	0	1	918	99.67	0.9
S. pombe	I	5479	1	286	5.22	0.83	1	5512	100.6	0.96
	II	4456	7	1823	40.91	0.71	1	4505	101.1	0.94
	II	2402	2	1064	44.3	0.8	1	3154	131.31	0.95

Supplementary References

1. Reisner, W., Pedersen, J. N. & Austin, R. H. DNA confinement in nanochannels: Physics and biological applications. *Reports on Progress in Physics* **75**, (2012).
2. Dvirnas, A. *et al.* DOGMA: de novo assembly of densely labelled optical DNA maps using a matrix profile approach. *PLoS One* **20**, e0335633- (2025).
