# Supplementary material for: Chromosome-scale assembly of yeast genomes using enzyme-free, dense optical genome mapping
Source: Nat Commun. 2026 Oct 1;17:10404. doi: 10.1038/s41467-026-78211-7 (PMC13631253; doi:10.1038/s41467-026-78211-7)
Supplement: Supplementary file 3 [file 41467_2026_78211_MOESM3_ESM.pdf]

nature portfolio

Peer Review File

Chromosome-scale assembly of yeast genomes using enzyme-free, dense optical genome mapping

Corresponding Author: Professor Fredrik Westerlund

This file contains all reviewer reports in order by version, followed by all author rebuttals in order by version.

Version 0:

Reviewer comments:

Reviewer #1

(Remarks to the Author)
The manuscript presents an interesting extension of the dense optical genome mapping assembly (DOGMA) framework from bacterial to eukaryotic genomes. The application to S. cerevisiae and S. pombe demonstrates the potential of dense OGM for analyzing large repetitive structures and chromosome-scale optical assemblies. In particular, the discussion of repeat-associated alignment ambiguity and long-range compositional signal trends represents an important consideration for dense optical mapping approaches. Importantly, the authors observed that repetitive barcode components can drive erroneous or overextended alignments between unrelated loci, and addressed this issue by masking repetitive regions prior to alignment and assembly. This repeat-aware alignment strategy represents a significant methodological advance for dense OGM analysis, particularly for repeat-rich eukaryotic genomes where repetitive optical signals can otherwise dominate global similarity metrics and compromise assembly accuracy.

However, several aspects of the manuscript would benefit from clarification and additional validation.

Major comments

1. Figure organization and readability

Figures 3 and 4 are difficult to follow because multiple distinct conceptual layers are combined into single composite figures. For example, Fig. 3 simultaneously addresses chromosome-spanning molecules, repetitive-region masking, long-range compositional trends, and ΔCC threshold optimization. Similarly, Fig. 4 combines repeat-aware assembly, SNR-based quality filtering, and final assembly examples. Separating these topics into more focused figures would substantially improve readability and strengthen the narrative flow.

2. The concept of long-range intensity trends is central to the manuscript but remains somewhat difficult to interpret mechanistically. Additional clarification would help readers understand:

- how these trends arise from large-scale GC/AT compositional domains,
- how they differ from local dense OGM features,
- and how they specifically influence global CC-based alignment ambiguity.

A schematic illustration decomposing local dense OGM features and low-frequency compositional trends may improve accessibility.

3. The term "bargroup" is not immediately intuitive, particularly in the context of dense continuous optical profiles rather than sparse barcode-like labeling. Consider replacing or supplementing this terminology with more standard concepts such as:
- DOGMA contig
- dense optical profile contig
- consensus DOGMA map
or other terminology that more directly reflects the continuous dense-profile nature of the data.

This may improve clarity for readers outside the immediate DOGMA framework.

4. The manuscript (Fig. 3 and Supplementary Fig. 3) shows that several strategies improve barcode-to-reference alignment specificity. However, it remains unclear how these improvements translate into better de novo assembly performance. In particular, chromosome-spanning molecule issues would not appear in the de novo assembly, and local alignment is already implemented as a core component of the DOGMA pipeline. Also, while ΔCC thresholding improves reference-based alignment accuracy, its contribution to bargroup formation or final assembly performance is unclear. A more explicit connection between the Fig.3 analyses and the actual DOGMA assembly process would strengthen the manuscript.

5. Figures i and ii in Fig. 3b and Fig. 4a are intended to show the effect of repeat-region masking, but it is unclear whether the same barcodes are used. If the barcodes in i and ii are the same, this should be more clearly indicated visually. If not, using the same barcodes for comparison would better demonstrate the effect of masking.

6. At line 264, the manuscript states that the similarity hotspots in Supplementary Fig. 4 correspond to the shared long-range signal features shown in Fig. 3c. However, this correspondence is difficult to interpret visually. For example, it is not obvious how the blue-box regions in Fig. 3c relate to the similarity hotspots shown in Supplementary Fig. 4.

7. There appears to be a discrepancy between the text description (lines 299-304) and the caption of Fig. 4b regarding the interpretation of the SNR-based bargroup scoring examples. Clarifying the relationship between panels i and ii and their corresponding SNR characteristics would improve readability.

8. The manuscript reports substantial extensions of repetitive regions, particularly:
- chromosome XII rDNA array in S. cerevisiae
- chromosome III terminal repeat structures in S. pombe

While the dense OGM data strongly suggest that repetitive regions may be underrepresented or collapsed in current reference assemblies, the current evidence remains primarily optical and inferential. Additional orthogonal experimental validation would substantially strengthen these conclusions.
In particular, PFGE/electrokaryotyping appears highly suitable for validating the proposed chromosome-scale size differences, especially for S. pombe chromosome III, where large terminal repeat expansions are proposed. If PFGE results are ambiguous, rare-cutter restriction analysis (e.g., NotI digestion followed by PFGE) could provide additional structural support and localization of the expanded regions.

9. It would be informative to compare assemblies generated before applying the introduced strategies (e.g., as in Fig. 5). Also, additional quantitative assembly metrics would help clarify how effectively these strategies improve assembly performance. For example, the visual impression of several assemblies, particularly chromosome XII in Fig. 5, appears substantially more dramatic than the quantitative description provided in the text. It would be helpful if the authors explicitly reported:
- estimated assembled chromosome lengths,
- estimated repeat-array sizes,
- how these values compare numerically with the reference assemblies.

This would help readers distinguish between schematic visualization effects and actual inferred size differences.

10. It would be helpful to include representative visualizations of the detected structural variations (SV), particularly at the locations described in the manuscript.

11. The discussion comparing dense OGM with sparse-label OGM is interesting and could be expanded further, particularly regarding which genomic structures are uniquely accessible by dense compositional profiling.

(Remarks on code availability)

Reviewer #2

(Remarks to the Author)
Complete de novo assembly of yeast genomes using enzyme-free, dense optical genome mapping

The manuscript presented by Leal-Garza et al describes a method for assembly of optical maps that are derived from DNA stained in a sequence-dependent manner, using a combination of YOYO-1 and netropsin. The general approach has been previously reported for optical mapping of bacterial genomes, which this work significantly extends to the yeast genomes. Given the ease with which samples can be prepared for mapping and the single-molecule, long-range nature of the information derived, I recommend this work is accepted for publication, subject to corrections as follows:

Line 64: “CB-based OGM exploits the competitive interaction between YOYO-1, a non-specific fluorescent DNA bis-intercalator, and netropsin…” To the best of my knowledge, there is no interaction between these two molecules, rather they compete for binding to DNA. Please rephrase.
Line 68: “…this method cannot resolve individual fluorophores, and hence sequence sites…” Resolving an individual

fluorophore does not lead to resolution of individual bases (if this is what 'sequence sites' refers to?) Please rephrase.
Figure 1c: In my copy the blue and grey are near impossible to resolve. Please recolour or increase the size of the image.
Line 149: For the reported mean coverage depths, what are the mean (equivalent) mapping rates of molecules? i.e. how much DNA do you need to image to generate these coverage depths?
Figure 2: I find the Figure confusing, particularly where the inset from panel a sits in panel b. Please reorganise.
Line 188: "Each of these properties can affect similarity scores and influence the grouping during assembly…" Please make a note that you show data to support this argument in the sections that follow. I don't see a definition of what constitutes a 'long-range intensity trend' in the text. This would be helpful, even if it is descriptive/qualitative.
Figure 3: Do the yeast centromeres not contain repetitive regions, as in the human genome? Could this not be the underlying cause of the low intensity for aligned barcodes?
Line 236: "…barcodes from distinct genomic loci exhibited high global similarity." I have no idea what 'high global similarity' is- please define or rephrase. Same comment as above on 'long-range intensity variation' in this paragraph. It is unclear to me what this is.
Line 250: "…experimental barcodes sharing similar long-range intensity trends…" Please define properly. What does 'intensity' refer to in this case? Simply the average fluorescence intensity of the aligned molecules?
Line 251: "including preferential placement at incorrect loci…" How do you define a correct/incorrect alignment?
Line 256: "…pose challenges for similarity-based alignment and assembly…" I think they pose a challenge for the approach you have adopted, specifically? Please rephrase.
Line 257: "…also provide direct insight into large-scale chromosomal organization…" This goes beyond what you show in the manuscript and is not supported by your data. Comparing mapping intensity to e.g. Hi-C data would be a way to support this argument.
Line 269: "… these findings indicate that structural properties intrinsic to eukaryotic genomes, rather than insufficient coverage…" There is no evidence to support this argument in the manuscript as written. Please remove or add supporting data. I would also recommend that the authors look at the GC content of the genome, on this scale and confirm that this doesn't underpin some of the observed long-range variation in intensity.
Line 274: "…QC procedures included stitching molecules spanning multiple imaging fields and masking barcode regions dominated by repetitive features…" This is not quality control, it's image processing. Please rephrase.
Can the authors also account for the fact that 'stitching molecules spanning multiple fields' has no impact on the mean molecule length?
Line 284: "…a ΔCC threshold to filter barcodes…" How is cross-correlation and the delta measured?
Line 287: How is the optimal ΔCC threshold identified? Justify that this is 'optimal'.
Line 314: What metrics are used to determine QC pass/fail and how were thresholds set?
Line 341: "…which omits the intensity of the first and last 6 pixels, assuming them to be uncertain." Surely this would lead to a shorter molecule, relative to the reference? Something not clear in the wording or logic.
Line 358, paragraph: How have the detected structural variations been validated? This must be confirmed using an orthogonal technology (e.g. PCR over an identified breakpoint with the reference).
Line 415: "…capturing continuous intensity profiles, that are orthogonal to sequence-based information, over hundreds of kilobases." The intensity profiles are wholly dependent on the underlying DNA sequence. Please rephrase ("sequencing"?)
Line 421: "…arises from YOYO-1 labeling of the DNA backbone" YOYO-1 doesn't label the DNA backbone. Please correct. Also Line 422.
Line 425: "already existing…" Delete 'already'.

(Remarks on code availability)

Version 1:

Reviewer comments:

Reviewer #1

(Remarks to the Author)
The revised manuscript is substantially clearer, and the authors have addressed many of the concerns raised in my previous review. In particular, the expanded explanation of long-range intensity trends, supported by the new Supplementary Fig. 4, and the addition of chromosome-level assembly metrics before and after application of the revised strategies in Supplementary Table 4 have improved the manuscript. The authors have also corrected several inconsistencies in the figures and improved the corresponding descriptions in the main text. I have some comments.

De novo nature of the chromosome-scale assemblies
The manuscript describes the final chromosome-scale assemblies as de novo. However, the workflow leading from the initially assembled bargroups to the final chromosome-scale assemblies remains unclear. The Results state that, following initial assembly, bargroups were aligned to the reference genomes and that "adjacent bargroups with consistent placements" were subsequently merged to generate chromosome-scale assemblies. This wording suggests that reference coordinates may have been used to select, order, or orient bargroups before the final merging step. In a fully de novo workflow, bargroup ordering and merging should be completed independently of the reference, with reference alignment performed only afterward for validation and visualization. The authors should clarify whether reference identity, position, order, or orientation was used at any stage in constructing the final chromosome-scale assemblies. If the reference influenced this process, the final step should be described as reference-assisted chromosome-scale reconstruction, and the title and central claims should be revised accordingly. Alternatively, the authors should demonstrate that the reported

chromosome-scale assemblies can be reconstructed without reference-guided placement.

Organization and interpretation of Fig. 3
The organization of Fig. 3 remains conceptually unclear. Panels a–c describe molecule, barcode, or genomic features affecting correlation-based alignment and assembly, whereas panel d presents the empirical selection of a ΔCC threshold for reference-alignment filtering. Because ΔCC is an analysis or filtering parameter rather than an intrinsic barcode or genomic feature, panel d is conceptually distinct from the other panels and may be more appropriate for the Supplementary Information. More importantly, the manuscript should clarify whether the ΔCC threshold is used only for reference-based alignment analysis or also to select barcodes entering de novo bargroup formation. If it is not part of the de novo assembly procedure, its presentation as a major assembly consideration should be reconsidered.

Minor comment
At line 173, the phrase “a complementary pattern” is ambiguous. If “complementary” is intended to imply that the depleted and enriched regions result from corresponding molecule misplacement, this relationship should be stated more explicitly.

(Remarks on code availability)

Reviewer #2

(Remarks to the Author)
I thank the authors for addressing the comments I had on the original manuscript and recommend this revised version for publication.

(Remarks on code availability)

Version 2:

Reviewer comments:

Reviewer #1

(Remarks to the Author)
The authors have adequately addressed all of my comments and questions. I have no further concerns.

(Remarks on code availability)

Open Access This Peer Review File is licensed under a Creative Commons Attribution 4.0 International License, which permits use, sharing, adaptation, distribution and reproduction in any medium or format, as long as you give appropriate

credit to the original author(s) and the source, provide a link to the Creative Commons license, and indicate if changes were made.
In cases where reviewers are anonymous, credit should be given to 'Anonymous Referee' and the source.
The images or other third party material in this Peer Review File are included in the article's Creative Commons license, unless indicated otherwise in a credit line to the material. If material is not included in the article's Creative Commons license and your intended use is not permitted by statutory regulation or exceeds the permitted use, you will need to obtain permission directly from the copyright holder.
To view a copy of this license, visit https://creativecommons.org/licenses/by/4.0/

Response to reviewers

Dear Dr. Fraser Andrews,

Thank you for reconsidering our manuscript for publication in Nature Communications. We thank the reviewers for the valuable feedback on our paper "Complete de novo assembly of yeast genomes using enzyme-free, dense optical genome mapping." We feel that addressing them has increased the readability and overall quality of our work. According to the editorial request we here resubmit the revised manuscript.

The point-by point replies to the reviewers' comments are included on the subsequent pages with reviewer comments in purple and our replies in black.

REVIEWER COMMENTS

Reviewer #1 (Remarks to the Author):

The manuscript presents an interesting extension of the dense optical genome mapping assembly (DOGMA) framework from bacterial to eukaryotic genomes. The application to S. cerevisiae and S. pombe demonstrates the potential of dense OGM for analyzing large repetitive structures and chromosome-scale optical assemblies. In particular, the discussion of repeat-associated alignment ambiguity and long-range compositional signal trends represents an important consideration for dense optical mapping approaches. Importantly, the authors observed that repetitive barcode components can drive erroneous or overextended alignments between unrelated loci, and addressed this issue by masking repetitive regions prior to alignment and assembly. This repeat-aware alignment strategy represents a significant methodological advance for dense OGM analysis, particularly for repeat-rich eukaryotic genomes where repetitive optical signals can otherwise dominate global similarity metrics and compromise assembly accuracy. However, several aspects of the manuscript would benefit from clarification and additional validation.

Major comments

1. Figure organization and readability

Figures 3 and 4 are difficult to follow because multiple distinct conceptual layers are combined into single composite figures. For example, Fig. 3 simultaneously addresses chromosome-spanning molecules, repetitive-region masking, long-range compositional trends, and ΔCC threshold optimization. Similarly, Fig. 4 combines repeat-aware assembly, SNR-based quality filtering, and final assembly examples. Separating these topics into more focused figures would substantially improve readability and strengthen the narrative flow.

We agree that the original presentation could be difficult to follow and have resized and revised the figures, captions, and corresponding text to strengthen the conceptual links between the individual panels and improve the narrative flow. We have retained the overall figure organization because each panel addresses a different aspect of a common methodological concept (Fig. 3: factors influencing correlation-based alignment and assembly; Fig. 4: assembly improvement and workflow). We believe that the revised presentation improves readability while preserving this conceptual organization.

2. The concept of long-range intensity trends is central to the manuscript but remains somewhat difficult to interpret mechanistically. Additional clarification would help readers understand:

- how these trends arise from large-scale GC/AT compositional domains,

- how they differ from local dense OGM features,

- and how they specifically influence global CC-based alignment ambiguity.

A schematic illustration decomposing local dense OGM features and low-frequency compositional trends may improve accessibility.

We thank the reviewer for this suggestion. We agree that the concept of long-range intensity trends required a clearer mechanistic explanation. We have expanded the description in the manuscript, introduced an explicit definition of long-range intensity trends, clarified their relationship to large-scale GC/AT compositional variation and local barcode features, and added a new Supplementary Fig. 4, which illustrates the decomposition of representative barcode profiles into low-frequency (long-range) and local components. The figure further demonstrates that these long-range trends increase correlation-based similarity between unrelated barcode regions, thereby contributing to alignment ambiguity (thus complicating assembly).

3. The term "bargroup" is not immediately intuitive, particularly in the context of dense continuous optical profiles rather than sparse barcode-like labeling. Consider replacing or supplementing this terminology with more standard concepts such as:

- DOGMA contig

- dense optical profile contig

- consensus DOGMA map

or other terminology that more directly reflects the continuous dense-profile nature of the data. This may improve clarity for readers outside the immediate DOGMA framework.

We agree that the term *bargroup* may not be immediately intuitive to readers unfamiliar with the DOGMA framework. Because this terminology was introduced in our previous work and is used consistently throughout the DOGMA pipeline, we have retained it for consistency. To improve

clarity, we now introduce the term more explicitly at its first occurrence, describing bargroups as optical mapping contigs before introducing the established DOGMA terminology.

4. The manuscript (Fig. 3 and Supplementary Fig. 3) shows that several strategies improve barcode-to-reference alignment specificity. However, it remains unclear how these improvements translate into better de novo assembly performance. In particular, chromosome-spanning molecule issues would not appear in the de novo assembly, and local alignment is already implemented as a core component of the DOGMA pipeline. Also, while ΔCC thresholding improves reference-based alignment accuracy, its contribution to bargroup formation or final assembly performance is unclear. A more explicit connection between the Fig.3 analyses and the actual DOGMA assembly process would strengthen the manuscript.

We appreciate the reviewer highlighting this important connection. Fig. 3 uses reference alignment as an interpretable framework to investigate sources of ambiguity that also affect *de novo* assembly. Because DOGMA constructs bargroups by ranking correlation-based similarities between barcodes, the same long-range intensity trends that reduce alignment specificity also reduce the specificity of barcode grouping. Also, the significance of a good match is determined by modelling a null distribution in DOGMA. While this null distribution is useful to discriminate between significant and non-significant matches between regular experimental barcodes, barcodes with long-range intensity trends will match to each other with higher scores, even if unrelated. We have clarified this relationship in the manuscript and now explicitly reference Supplementary Fig. 4, which demonstrates that long-range intensity trends systematically increase pairwise correlation scores and therefore increase the likelihood of ambiguous barcode grouping.

Regarding the chromosome-spanning molecules, we agree that they do not directly affect barcode grouping in the same manner as repetitive regions or long-range intensity trends. We have revised the manuscript to clarify that this section serves two purposes: (i) demonstrating that dense OGM routinely captures complete yeast chromosomes within single molecules, and (ii) motivating the use of local alignment during comparison of assembled bargroups to the reference, where just like chromosome-spanning molecules, any bargroup longer than the reference would otherwise be incorrectly penalized (or mapped elsewhere) by full-length alignment. We have also modified the introductory text to distinguish this from the subsequent sections describing factors that directly influence similarity-based assembly.

5. Figures i and ii in Fig. 3b and Fig. 4a are intended to show the effect of repeat-region masking, but it is unclear whether the same barcodes are used. If the barcodes in i and ii are the same, this should be more clearly indicated visually. If not, using the same barcodes for comparison would better demonstrate the effect of masking.

We thank the reviewer for this suggestion. In Fig. 3b, panels i and ii do indeed show the same barcodes before and after repeat-region masking. This is shown by color coding. To make this clearer, we have revised the figure legend to explicitly state that the same barcodes are shown in both panels.

In contrast, the examples shown in Fig. 4a do not contain the same constituent barcodes. Repeat-region masking alters barcode similarity scores and therefore affects which (and how many) barcodes that are grouped together during assembly. As a result, bargroups assembled with and without masking may contain different barcode members and are not directly comparable on a barcode-by-barcode basis. We have clarified that the constituent barcodes are not the same in the figure legend.

6. At line 264, the manuscript states that the similarity hotspots in Supplementary Fig. 4 correspond to the shared long-range signal features shown in Fig. 3c. However, this correspondence is difficult to interpret visually. For example, it is not obvious how the blue-box regions in Fig. 3c relate to the similarity hotspots shown in Supplementary Fig. 4.

We agree that the correspondence between the similarity hotspots shown in the old Supplementary Fig. 4 (now Supplementary Fig. 5) and the regions exhibiting pronounced long-range intensity trends in Fig. 3c were not sufficiently clear in the original manuscript. To improve readability, we have revised the text to describe this relationship more cautiously and avoid implying a direct one-to-one correspondence. In addition, the genomic regions highlighted in Fig. 3c are now also indicated in Supplementary Fig. 5, making the qualitative overlap between the two analyses easier to identify.

7. There appears to be a discrepancy between the text description (lines 299-304) and the caption of Fig. 4b regarding the interpretation of the SNR-based bargroup scoring examples. Clarifying the relationship between panels i and ii and their corresponding SNR characteristics would improve readability.

We thank the reviewer for identifying this inconsistency. This discrepancy arose from an error in the in-text referencing of panels b.i and b.ii in Fig. 4b. The figure caption is correct, and the mismatch has been corrected in the main text. Specifically, the references to panels b.i and b.ii have been swapped to ensure consistency between the textual description and the figure panels. This correction does not affect the interpretation of the SNR-based bargroup scoring metric or the conclusions drawn from these examples.

8. The manuscript reports substantial extensions of repetitive regions, particularly:

- chromosome XII rDNA array in S. cerevisiae

- chromosome III terminal repeat structures in S. pombe

While the dense OGM data strongly suggest that repetitive regions may be underrepresented or collapsed in current reference assemblies, the current evidence remains primarily optical and inferential. Additional orthogonal experimental validation would substantially strengthen these conclusions.

In particular, PFGE/electrokaryotyping appears highly suitable for validating the proposed chromosome-scale size differences, especially for S. pombe chromosome III, where large terminal repeat expansions are proposed. If PFGE results are ambiguous, rare-cutter restriction analysis (e.g., NotI digestion followed by PFGE) could provide additional structural support and localization of the expanded regions.

We thank the reviewer for this suggestion. We agree that PFGE is a valuable approach for assessing chromosome-scale variation in repetitive regions. While we did not perform new PFGE experiments as part of this study, previous PFGE studies have documented size variability in both the *S. cerevisiae* chromosome XII rDNA locus and the *S. pombe* chromosome III terminal rDNA arrays. In particular, Pasero and Marilley (1993) (ref 29) estimated approximately 115 rDNA repeat units distributed across the two terminal arrays and reported chromosome-size variation due to changes in rDNA copy number in *S. pombe*. These observations are consistent with the large terminal repeat structures recovered in our DOGMA assemblies. We have expanded the discussion of these prior PFGE results in the revised manuscript and clarified the relationship between our findings and previous experimental observations.

9. It would be informative to compare assemblies generated before applying the introduced strategies (e.g., as in Fig. 5). Also, additional quantitative assembly metrics would help clarify how effectively these strategies improve assembly performance. For example, the visual impression of several assemblies, particularly chromosome XII in Fig. 5, appears substantially more dramatic than the quantitative description provided in the text. It would be helpful if the authors explicitly reported:

- estimated assembled chromosome lengths,

- estimated repeat-array sizes,

- how these values compare numerically with the reference assemblies.

This would help readers distinguish between schematic visualization effects and actual inferred size differences.

We thank the reviewer for this helpful suggestion. We agree that a quantitative comparison complements the schematic representation in Fig. 5. Since the number and identity of bargroups produced by the algorithm changes depending on the masking /filtering done to the bargroups fed, it is not meaningful to do a one-to-one comparison of bargroups before and after the introduced strategies. Instead, we decided to align all the resulting bargroups to the reference genome and do a chromosome level comparison.

We have therefore added Supplementary Table 4, which summarizes chromosome-level assembly metrics before and after introducing the strategies described in this work. For each reference chromosome, the table reports the number of assembled bargroups aligning to that chromosome, the inferred assembled length (assuming alignment is correct), the percentage of reference

coverage, and the mean alignment score (CC). This provides a quantitative comparison of the assembly outputs and complements the visual examples shown in Fig. 5. We have also expanded the description in the Results to direct readers to Supplementary Table 3. Repeat-array sizes are not meaningful in the bargroups generated previous to the introduced strategies, since repetitive bargroups were frequently grouped with non-repetitive barcodes, dimming the repetitive signal.

10. It would be helpful to include representative visualizations of the detected structural variations (SV), particularly at the locations described in the manuscript.

We thank the reviewer for this suggestion. To better illustrate the regions discussed in the manuscript, we have added Supplementary Fig. 7, which presents representative examples of the discrepancies (originally described as SVs) observed between the DOGMA assemblies and the S288c reference assembly in chromosomes III and VIII.

Following further investigation, we compared these regions against independently generated long-read assemblies of S288c (ref. 30). In both cases, theoretical barcodes generated from the long-read assemblies showed improved agreement with the DOGMA assemblies relative to the R64 reference assembly. Based on this additional evidence, we revised the manuscript text to describe these regions as assembly discrepancies relative to the reference rather than real biological SVs.

11. The discussion comparing dense OGM with sparse-label OGM is interesting and could be expanded further, particularly regarding which genomic structures are uniquely accessible by dense compositional profiling.

We have expanded the Discussion to more explicitly contrast sparse motif-based OGM with dense CB-based compositional profiling, highlighting the classes of genomic features that become accessible due to continuous intensity measurement, including extended compositional domains (shown as long-range intensity trend regions) and repetitive or low-motif-density regions that are not directly resolved in sparse labeling schemes.

Reviewer #2 (Remarks to the Author):

Complete de novo assembly of yeast genomes using enzyme-free, dense optical genome mapping

The manuscript presented by Leal-Garza et al describes a method for assembly of optical maps that are derived from DNA stained in a sequence-dependent manner, using a combination of YOYO-1 and netropsin. The general approach has been previously reported for optical mapping of bacterial genomes, which this work significantly extends to the yeast genomes. Given the ease with which samples can be prepared for mapping and the single-molecule, long-range nature of the information derived, I recommend this work is accepted for publication, subject to corrections as follows:

1. Line 64: "CB-based OGM exploits the competitive interaction between YOYO-1, a non-specific fluorescent DNA bis-intercalator, and netropsin…" To the best of my knowledge, there is no interaction between these two molecules, rather they compete for binding to DNA. Please rephrase.

We thank the reviewer for pointing this out. We agree that our wording was confusing, since YOYO-1 and netropsin do not interact directly, but instead compete for binding to DNA. We have revised the text accordingly to improve accuracy and clarity.

2. Line 68: "…this method cannot resolve individual fluorophores, and hence sequence sites…" Resolving an individual fluorophore does not lead to resolution of individual bases (if this is what 'sequence sites' refers to?) Please rephrase.

We agree that the original phrasing was misleading, as the method does not provide (nor aims to provide) base-level resolution of the sequence. We have revised the sentence to remove this implication and clarify that the approach generates continuous, information-dense intensity profiles rather than resolving individual fluorophores or sequence positions.

3. Figure 1c: In my copy the blue and grey are near impossible to resolve. Please recolour or increase the size of the image.

We thank the reviewer for his observation. For the sake of clarity, in Fig. 1c, colors have been modified to blue and black, linewidth has been increased. Panel 1b was also recolored and resized for consistency and readability.

4. Line 149: For the reported mean coverage depths, what are the mean (equivalent) mapping rates of molecules? i.e. how much DNA do you need to image to generate these coverage depths?

Coverage depths were calculated from molecules that passed quality control and were successfully aligned to the reference genome, and therefore reflect the usable fraction of the dataset contributing to assembly. We have clarified this definition in the revised manuscript.

5. Figure 2: I find the Figure confusing, particularly where the inset from panel a sits in panel b. Please reorganise.

We thank the reviewer for this suggestion. Fig. 2 has been reorganized to improve clarity and ensure correct placement of the insets within both panels.

6. Line 188: "Each of these properties can affect similarity scores and influence the grouping during assembly…" Please make a note that you show data to support this argument in the sections that follow. I don't see a definition of what constitutes a 'long-range intensity trend' in the text. This would be helpful, even if it is descriptive/qualitative.

We thank the reviewer for pointing this out. We have now introduced an explicit definition of long-range intensity trends when the concept is first introduced, clarified that they correspond to low-frequency variations in the continuous fluorescence intensity profile spanning hundreds of kilobases, and added a forward reference to the analyses presented later in the manuscript. Additional mechanistic explanation is provided in the new Supplementary Fig. 4.

7. Figure 3: Do the yeast centromeres not contain repetitive regions, as in the human genome? Could this not be the underlying cause of the low intensity for aligned barcodes?

The low-intensity regions shown in Fig. 3c correspond to theoretical barcode profiles (meaning that they are modeled from the reference genome) rather than experimental barcode coverage. Although yeast centromeres contain repetitive DNA, the observed reduction in fluorescence intensity primarily reflects local sequence composition (AT-richness) rather than a direct measure of repeat content.

8. Line 236: "…barcodes from distinct genomic loci exhibited high global similarity." I have no idea what 'high global similarity' is- please define or rephrase. Same comment as above on 'long-range intensity variation' in this paragraph. It is unclear to me what this is.

We agree that the original wording was ambiguous. We now explicitly refer to high Pearson correlation scores and define long-range intensity trends earlier in the manuscript.

9. Line 250: "…experimental barcodes sharing similar long-range intensity trends…" Please define properly. What does 'intensity' refer to in this case? Simply the average fluorescence intensity of the aligned molecules?

We agree that the terminology required clarification. Throughout the revised manuscript, we have now made sure that we say "intensity profiles" rather than "intensity" on its own, since we refer explicitly to the position-dependent fluorescence intensity profile measured along the DNA molecule rather than the average fluorescence intensity of the molecule. The new Supplementary Fig. 4 illustrates this concept by decomposing representative barcode profiles into low-frequency (long-range) and local intensity components.

10. Line 251: "including preferential placement at incorrect loci…" How do you define a correct/incorrect alignment?

We agree that the wording was imprecise for experimental barcodes, whose true genomic origin is not independently known. We have therefore replaced "incorrect loci" with a description based on the observed alignment ambiguity, quantified as a small difference between the highest- and second-highest-scoring alignments (ΔCC). We also clarify in the manuscript that ΔCC is defined as the difference between the best and second-best Pearson correlation scores.

11. Line 256: "…pose challenges for similarity-based alignment and assembly…" I think they pose a challenge for the approach you have adopted, specifically? Please rephrase.

We have revised the text to clarify that long-range intensity trends specifically complicate correlation-based alignment and assembly of dense barcodes, rather than referring to a general effect. The sentence has been rephrased accordingly.

12. Line 257: "…also provide direct insight into large-scale chromosomal organization…" This goes beyond what you show in the manuscript and is not supported by your data. Comparing mapping intensity to e.g. Hi-C data would be a way to support this argument.

We thank the reviewer for this comment. We agree that the phrase "large-scale chromosomal organization" could be interpreted as implying information about higher-order or spatial chromosome organization, which was not the intended meaning. We have revised the text to more precisely describe these signals in terms of chromosome-scale compositional sequence variation captured by dense OGM.

13. Line 269: "… these findings indicate that structural properties intrinsic to eukaryotic genomes, rather than insufficient coverage…" There is no evidence to support this argument in the manuscript as written. Please remove or add supporting data. I would also recommend that the authors look at the GC content of the genome, on this scale and confirm that this doesn't underpin some of the observed long-range variation in intensity.

We agree that the original wording was too broad and that this statement required additional support. We have revised the manuscript to avoid referring to "structural properties intrinsic to eukaryotic genomes" and instead describe the specific genome-scale sequence features observed in this study, namely repetitive regions and large-scale compositional variation. We also added a new Supplementary Fig. 4, which illustrates how long-range intensity trends arising from large-scale compositional sequence variation increase correlation-based similarity and alignment ambiguity. It is important to note that both, Fig. 3c and the new Supplementary Fig. 4 look at long-range intensity trends arising from the intensity profile of the theoretical barcodes derived from the sequence of the reference assemblies. This implies that, as suggested, the gradual increase/decrease in GC content is the source of the long-range variation in intensity. This was done using the reference barcode to ensure that we have control of the loci and to avoid interpreting what could be an illumination artifact.

14. Line 274: "…QC procedures included stitching molecules spanning multiple imaging fields and masking barcode regions dominated by repetitive features…" This is not quality control, it's image processing. Please rephrase. Can the authors also account for the fact that 'stitching molecules spanning multiple fields' has no impact on the mean molecule length?

We thank the reviewer for this observation. We agree that stitching molecules spanning multiple imaging fields is a preprocessing step rather than a QC procedure. The manuscript has been revised

to clearly distinguish barcode preprocessing (including stitching and repetitive-region masking) from QC filtering.

Stitching is performed only when two barcode segments are identified as originating from the same DNA molecule based on overlap of (at least 130px) their barcode profiles and acquisition metadata. In these cases, overlapping segments are merged into a single reconstructed barcode, reducing the number of fragments while extending the corresponding molecule representation. Because this procedure affects only a small fraction of molecules, the overall mean molecule length remains largely unchanged. This clarification has been added to the Supplementary Methods.

15. Line 284: "…a ΔCC threshold to filter barcodes…" How is cross-correlation and the delta measured?

We agree that this terminology can be confusing. Pearson correlation is defined in the Methods as the normalized cross-correlation between barcode intensity profiles. ΔCC is defined as the difference between the highest and second-highest correlation scores obtained during alignment. We have clarified this definition in the revised manuscript.

16. Line 287: How is the optimal ΔCC threshold identified? Justify that this is 'optimal'.

We agree that "optimal" overstated the result. The revised manuscript now describes the selected ΔCC threshold as an empirically chosen compromise between improved specificity and barcode retention, as illustrated in Fig. 3d and Supplementary Fig. 3.

17. Line 314: What metrics are used to determine QC pass/fail and how were thresholds set?

The quality-control parameters used in this study were largely adopted from our previously established dense OGM pipeline (ref. 14), where they are better described and validated.

In particular, shrink-filtering parameters were chosen to exclude molecules that had not reached equilibrium extension in nanochannels, ensuring that barcode features could be reliably resolved and locally aligned without overlap-induced distortion. In addition, a minimum effective barcode length threshold (150 px, corresponding to ~150 kbp) was applied following established practice in optical genome mapping workflows to ensure sufficient feature density for robust alignment and comparison.

These criteria are described in detail in the Methods and Supplementary Methods, where we now explicitly clarify their origin and role within the pipeline.

18. Line 341: "…which omits the intensity of the first and last 6 pixels, assuming them to be uncertain." Surely this would lead to a shorter molecule, relative to the reference? Something not clear in the wording or logic.

We thank the reviewer for pointing out that the original wording was unclear. The trimming operation is applied to both theoretical and experimental barcodes; however, its effect is not necessarily equivalent. In theoretical barcodes, the removed pixels correspond to defined positions

within the modeled barcode and may reduce the represented sequence length near the barcode boundaries. In contrast, the ends of experimental barcodes are determined through molecule detection and may not correspond precisely to the physical DNA boundaries. Consequently, trimming the experimental barcodes does not necessarily remove an equivalent amount of genomic sequence. We have revised the text to clarify this distinction and to avoid implying a definitive explanation for the observed terminal extensions.

19. Line 358, paragraph: How have the detected structural variations been validated? This must be confirmed using an orthogonal technology (e.g. PCR over an identified breakpoint with the reference).

We thank the reviewer for this comment. We understand that calling these "structural variations" might have been an overstatement. The apparent SVs reported in the original manuscript were identified through manual inspection of alignments between the DOGMA assemblies and the S288c reference genome. Since SV detection is not yet integrated into the DOGMA assembly workflow, these observations were initially interpreted conservatively as potential insertions relative to the reference assembly. To further investigate these regions, we compared our assemblies against publicly available long-read assemblies (ref 30). In both chromosome III and chromosome VIII, the theoretical barcodes generated from the long-read assemblies showed substantially improved agreement with our DOGMA assemblies relative to the R64 reference assembly. These observations suggest that the discrepancies may arise from missing, collapsed, or otherwise unresolved regions in the current reference assembly rather than representing novel strain-specific structural variants. We have therefore revised the manuscript accordingly and added Supplementary Fig. 7 to illustrate these comparisons.

20. Line 415: "…capturing continuous intensity profiles, that are orthogonal to sequence-based information, over hundreds of kilobases." The intensity profiles are wholly dependent on the underlying DNA sequence. Please rephrase ("sequencing"?)

As the reviewer comments, the continuous intensity profiles can even be derived from the sequence, so there is no way of calling them orthogonal. The sentence was rephrased to describe better in which way the information from this technique complements sequencing information.

21. Line 421: "…arises from YOYO-1 labeling of the DNA backbone" YOYO-1 doesn't label the DNA backbone. Please correct. Also Line 422.

We agree with this minor edit, while the common jargon is to say that YOYO-1 is used to label the DNA backbone (meaning to locate DNA without sequence specificity), YOYO-1 is a bisintercalator (which we describe in the text earlier). The sentence has been rephrased to avoid technical errors.

22. Line 425: "already existing…" Delete 'already'.

We agree with this minor edit, the sentence has been rephrased to be clearer.

REVIEWER COMMENTS

Reviewer #1 (Remarks to the Author):

The revised manuscript is substantially clearer, and the authors have addressed many of the concerns raised in my previous review. In particular, the expanded explanation of long-range intensity trends, supported by the new Supplementary Fig. 4, and the addition of chromosome-level assembly metrics before and after application of the revised strategies in Supplementary Table 4 have improved the manuscript. The authors have also corrected several inconsistencies in the figures and improved the corresponding descriptions in the main text. I have some comments.

We sincerely thank the reviewer for the careful and constructive evaluation of our manuscript throughout both rounds of review. We greatly appreciate the thoughtful comments and suggestions, which have helped us clarify the methodology, strengthen the interpretation of our results, and improve the overall presentation of the manuscript.

De novo nature of the chromosome-scale assemblies

The manuscript describes the final chromosome-scale assemblies as de novo. However, the workflow leading from the initially assembled bargroups to the final chromosome-scale assemblies remains unclear. The Results state that, following initial assembly, bargroups were aligned to the reference genomes and that “adjacent bargroups with consistent placements” were subsequently merged to generate chromosome-scale assemblies. This wording suggests that reference coordinates may have been used to select, order, or orient bargroups before the final merging step. In a fully de novo workflow, bargroup ordering and merging should be completed independently of the reference, with reference alignment performed only afterward for validation and visualization. The authors should clarify whether reference identity, position, order, or orientation was used at any stage in constructing the final chromosome-scale assemblies. If the reference influenced this process, the final step should be described as reference-assisted chromosome-scale reconstruction, and the title and central claims should be revised accordingly. Alternatively, the authors should demonstrate that the reported chromosome-scale assemblies can be reconstructed without reference-guided placement.

We thank the reviewer for identifying this ambiguity in our description of the assembly workflow. We agree that our previous wording did not sufficiently distinguish the initial OGM de novo assembly from the subsequent refinement used to obtain chromosome-scale assemblies in the earlier parts of the paper, but rather only in the later section of the results.

To clarify our pipeline: The initial assembly of barcodes into bargroups was performed using the DOGMA framework without reference-guided placement. Following this assembly step, some overlapping bargroups remained unresolved. We subsequently used local alignment of the assembled bargroups to the corresponding reference genomes to identify unresolved bargroup joins with consistent genomic placement and to guide their refinement into chromosome-scale

assemblies. Thus, reference information was not used to generate the initial bargroups, but was subsequently used to assist in the resolution of remaining assembly joins.

In addition, reference alignment served a different purpose later in the workflow. We used the reference-aligned assembly to identify two local breaks in contiguity and examined high-confidence barcodes with strong reference matches and low alignment ambiguity that had not been incorporated into bargroups. These molecules were used to "repair" the local contiguity breaks as described in the Results, and this procedure is now also better described in the Methods.

We agree that the final chromosome-scale reconstructions should not be described as entirely reference-independent. We have therefore revised the manuscript to explicitly describe this step as reference-assisted refinement and clarified the distinct roles of reference alignment in assembly refinement, local contiguity repair, and subsequent assembly validation and structural comparison. We have also adjusted the title to:

Chromosome-scale assembly of yeast genomes using enzyme-free, dense optical genome mapping

Organization and interpretation of Fig. 3

The organization of Fig. 3 remains conceptually unclear. Panels a–c describe molecule, barcode, or genomic features affecting correlation-based alignment and assembly, whereas panel d presents the empirical selection of a ΔCC threshold for reference-alignment filtering. Because ΔCC is an analysis or filtering parameter rather than an intrinsic barcode or genomic feature, panel d is conceptually distinct from the other panels and may be more appropriate for the Supplementary Information. More importantly, the manuscript should clarify whether the ΔCC threshold is used only for reference-based alignment analysis or also to select barcodes entering de novo bargroup formation. If it is not part of the de novo assembly procedure, its presentation as a major assembly consideration should be reconsidered.

We thank the reviewer for pointing out the conceptual distinction between the features presented in Fig. 3a–c and the empirical ΔCC analysis previously presented in Fig. 3d. We agree that the ΔCC analysis is more appropriately presented separately, together with the reference alignment for which it is used. We have therefore moved the former panel 3d to Supplementary Fig. 3c and revised the corresponding text to clarify its purpose.

Specifically, the purpose of the reference alignment is to assess whether the strategies applied account for the non-uniformity of the coverage. The local barcode-to-reference alignment and repeat masking strategies used in this analysis address chromosome-spanning molecules and repetitive regions, respectively, while the ΔCC analysis was used to assess residual alignment ambiguity that could arise in part from long-range intensity trends. The ΔCC threshold was not used for barcode selection during de novo bargroup formation. During de novo assembly, the influence of long-range intensity trends on correlation-based grouping was instead addressed using the SNR-based bargroup scoring metric described in the manuscript. We used ΔCC as a proxy during reference-based analysis since the SNR metric can only be applied until after the bargroups

are formed, due to how it is defined. The ΔCC threshold was subsequently also used to select for strongly matching barcodes with low alignment ambiguity used for the analysis and repair of the local contiguity breaks described in the manuscript. The main text has been modified to better reflect these distinct uses of the reference alignment and ΔCC threshold.

Minor comment

At line 173, the phrase "a complementary pattern" is ambiguous. If "complementary" is intended to imply that the depleted and enriched regions result from corresponding molecule misplacement, this relationship should be stated more explicitly.

We agree that the term "complementary pattern" was ambiguous in this context. We have replaced this wording with "an additional source of barcode misplacement" and made minor revisions to the surrounding text to clarify the relationship between the observed coverage patterns and the different mechanisms that can lead to barcode misplacement with our alignment method.

Reviewer #2 (Remarks to the Author):

I thank the authors for addressing the comments I had on the original manuscript and recommend this revised version for publication.

We thank the reviewer for their careful evaluation of the revised manuscript and for their positive assessment. We greatly appreciate the constructive feedback provided throughout the review process, which has helped us further improve the manuscript.
